# ROS Regulation During Abiotic Stress Responses in Crop Plants

**DOI:** 10.3389/fpls.2015.01092

**Published:** 2015-12-08

**Authors:** Jun You, Zhulong Chan

**Affiliations:** Key Laboratory of Plant Germplasm Enhancement and Specialty Agriculture, Wuhan Botanical Garden, Chinese Academy of SciencesWuhan, China

**Keywords:** crop plants, transcription factors, reactive oxygen species, abiotic stress, antioxidative enzymes, gene regulation

## Abstract

Abiotic stresses such as drought, cold, salt and heat cause reduction of plant growth and loss of crop yield worldwide. Reactive oxygen species (ROS) including hydrogen peroxide (H_2_O_2_), superoxide anions (O_2_^•-^), hydroxyl radical (OH•) and singlet oxygen (^1^O_2_) are by-products of physiological metabolisms, and are precisely controlled by enzymatic and non-enzymatic antioxidant defense systems. ROS are significantly accumulated under abiotic stress conditions, which cause oxidative damage and eventually resulting in cell death. Recently, ROS have been also recognized as key players in the complex signaling network of plants stress responses. The involvement of ROS in signal transduction implies that there must be coordinated function of regulation networks to maintain ROS at non-toxic levels in a delicate balancing act between ROS production, involving ROS generating enzymes and the unavoidable production of ROS during basic cellular metabolism, and ROS-scavenging pathways. Increasing evidence showed that ROS play crucial roles in abiotic stress responses of crop plants for the activation of stress-response and defense pathways. More importantly, manipulating ROS levels provides an opportunity to enhance stress tolerances of crop plants under a variety of unfavorable environmental conditions. This review presents an overview of current knowledge about homeostasis regulation of ROS in crop plants. In particular, we summarize the essential proteins that are involved in abiotic stress tolerance of crop plants through ROS regulation. Finally, the challenges toward the improvement of abiotic stress tolerance through ROS regulation in crops are discussed.

## Introduction

Abiotic stress conditions such as drought, heat, or salinity affect plant growth and reduce agricultural production worldwide. These reductions result from climate change and the freshwater-supply shortage as well as the simultaneous occurrence of different abiotic stresses (Mittler and Blumwald, 2010; Hu and Xiong, 2014). To meet the demands of food security in the face of an increasing world population and environmental challenge, scientists envisage a crucial need for a “second green revolution” to enhance crop yield and yield stability under non-optimal and adverse growing conditions by a combination of approaches based on the recent advances in genomic research (Zhang, 2007; Eckardt et al., 2009).

To cope with adverse conditions, plants have evolved a range of physiological and metabolic responses by activation of a great many of stress-responsive genes and synthesis of diverse functional proteins through a complex signal transduction network, so as to confer tolerance to the environmental stresses (Hirayama and Shinozaki, 2010). Reactive oxygen species (ROS), including hydrogen peroxide (H_2_O_2_), superoxide radical (O_2_^•-^), hydroxyl radical (OH•) and singlet oxygen (^1^O_2_) etc., resulting from excitation or incomplete reduction of molecular oxygen, are harmful by-products of basic cellular metabolism in aerobic organisms (Apel and Hirt, 2004; Miller et al., 2010). Besides the toxicity of ROS, ROS are also considered to be signaling molecules that regulate plant development, biotic and abiotic stress responses (Apel and Hirt, 2004; Mittler et al., 2004). Many excellent reviews have focused on ROS metabolism (Apel and Hirt, 2004; Noctor et al., 2014), ROS sensory and signaling networks (Miller et al., 2010; Suzuki et al., 2012; Baxter et al., 2014), as well as the cross-talk with other signaling molecules function in developmental and stress response processes (Suzuki et al., 2012; Noctor et al., 2014). However, most of these reviews provided an overall retrospective for model plant *Arabidopsis*. Gill and Tuteja (2010) reviewed enzymatic and non-enzymatic antioxidants and their roles in abiotic stress tolerance of crop plants. However, the regulation mechanism of the antioxidant system and the key components involved in ROS regulation and abiotic stress tolerance have not yet been summarized in crop plants. In this review, we provide an overview of current knowledge about ROS homeostasis regulation in crop plants. In particular, the genes that have been characterized in ROS homeostasis regulation affecting abiotic stress resistance in crop plants were summarized.

## Ros Homeostasis In Plant

The evolution of aerobic metabolic processes such as respiration and photosynthesis unavoidably led to the production of ROS in mitochondria, chloroplast, and peroxisome (Apel and Hirt, 2004; Gill and Tuteja, 2010). Under optimal growth conditions, intracellular ROS are mainly produced at a low level in organelles. However, ROS are dramatically acclimated during stress. Under abiotic stress condition, limitation of CO_2_ uptake, caused by stress-induced stomatal closure, favors photorespiratory production of H_2_O_2_ in the peroxisome and production of superoxide and H_2_O_2_ or singlet oxygen by the overreduced photosynthetic electron transport chain (Apel and Hirt, 2004; Noctor et al., 2014). In addition to organelles, plasma membrane together with apoplast is the main site for ROS generation in response to endogenous signals and exogenous environmental stimuli. Several types of enzymes, such as NADPH oxidases, amine oxidases, polyamine oxidases, oxalate oxidases, and a large family of class III peroxidases, that localized at the cell surface or apoplast are contributed to production of apoplast ROS (Apel and Hirt, 2004; Cosio and Dunand, 2009; Gill and Tuteja, 2010).

Overproduction of ROS caused by stress conditions in plant cells is highly reactive and toxic to proteins, lipids, and nucleic acid which ultimately results in cellular damage and death (Gill and Tuteja, 2010). On the other hand, the increased production of ROS during stresses also thought to act as signals for the activation of stress response pathways (Baxter et al., 2014). Plants have evolved an efficient enzymatic and non-enzymatic antioxidative system to protect themselves against oxidative damage and fine modulation of low levels of ROS for signal transduction.

ROS-scavenging enzymes of plants include superoxide dismutase (SOD), ascorbate peroxidase (APX), catalase (CAT), glutathione peroxidase (GPX), monodehydroascorbate reductase (MDHAR), dehydroascorbate reductase (DHAR), glutathione reductase (GR), glutathione *S*-transferase (GST), and peroxiredoxin (PRX). These antioxidant enzymes are located in different sites of plant cells and work together to detoxify ROS. SOD acts as the first line of defense converting O_2_^•-^ into H_2_O_2_. CAT, APX, and GPX then detoxify H_2_O_2_. In contrast to CAT, APX requires an ascorbic acid (AsA) and/or a glutathione (GSH) regenerating cycle involved MDHAR, DHAR, and GR. GPX, GST, and PRX reduce H_2_O_2_ and organic hydroperoxides through ascorbate-independent thiol-mediated pathways using GSH, thioredoxin (TRX) or glutaredoxin (GRX) as nucleophile (Dietz et al., 2006; Meyer et al., 2012; Noctor et al., 2014). Non-enzymatic antioxidants include GSH, AsA, carotenoids, tocopherols, and flavonoids are also crucial for ROS homeostasis in plant (Gill and Tuteja, 2010). Besides traditional enzymatic and non-enzymatic antioxidants, increasing evidences indicated that soluble sugars, including disaccharides, raffinose family oligosaccharides and fructans, have a dual role with respect to ROS (Couee et al., 2006; Keunen et al., 2013). Soluble sugars were directly linked with the production rates of ROS by regulation ROS producing metabolic pathways, such as mitochondrial respiration or photosynthesis. Conversely, they also feed NADPH-producing metabolism to participate in antioxidative processes (Couee et al., 2006).

In addition to the antioxidative system, avoiding ROS production by alleviating the effects of stresses on plant metabolism may also be important for keeping ROS homeostasis. Alternative oxidases (AOX) can prevent the excess generation of ROS in the electron transport chains of mitochondria (Maxwell et al., 1999). By diverting electrons flowing through electron-transport chains, AOX can decrease the possibility of electron leaking to O_2_ to generate O_2_^•-^. Other mechanisms, such as leaf movement and curling, photosynthetic apparatus rearranging, may also represent an attempt to avoid the over-reduction of ROS by balancing the amount of energy absorbed by the plant with the availability of CO_2_ (Mittler, 2002).

## Regulation Of Nadph Oxidases In Crop Plants

Plant NADPH oxidases, also known as respiratory burst oxidase homologs (RBOHs), are the most studied enzymatic source of ROS. Plant RBOHs have cytosolic FAD- and NADPH-binding domains in the C-terminal region, and transmembrane domains that correspond to those in mammalian NADPH oxidases (Suzuki et al., 2011). In addition, plant RBOHs have a cytosolic N-terminal extension contains regulatory regions such as calcium-binding EF-hands and phosphorylation target sites that are important for the function and regulation of the plant NADPH oxidases (Oda et al., 2010; Suzuki et al., 2011). Increasing evidence demonstrated NADPH oxidases as key signaling nodes in the ROS regulation network of plants integrating numerous signal transduction pathways with ROS signaling and mediating multiple important biological processes, including cell growth and plant development, abiotic stress response and adaptation, plant–microbe pathogenic and symbiotic interactions (Torres and Dangl, 2005; Suzuki et al., 2011; Marino et al., 2012). Numerous studies have uncovered several regulatory mechanisms of plant NADPH oxidases in *Arabidopsis*, which involved various signaling components including protein phosphorylation, Ca^2+^, CDPKs, and phospholipase Dα1 (PLDα1) (Baxter et al., 2014). Ca^2+^ regulates NADPH oxidase-dependent ROS production by binding directly to the EF-hand motif in the N terminus of RBOH protein and/or regulating Ca^2+^-dependent phosphorylation medicated by CDPK (Ogasawara et al., 2008; Dubiella et al., 2013). RBOHs were also found to be phosphorylated by SnRK2 protein kinase OPEN STOMATA 1 (OST1) during ABA-dependent stomatal closure (Sirichandra et al., 2009).

Functions and regulatory mechanisms of several RBOH proteins were investigated in crops. The activity of NADPH oxidase was increased by drought, and exhibited high-temperature stability and an alkaline-philic feature, suggesting its important role in response to drought stress (Duan et al., 2009). Treatment with ABA and Ca^2+^ also considerably induced the activity of NADPH oxidase in leaves of maize seedlings (Jiang and Zhang, 2002a, 2003). Nine NADPH oxidase (RBOH) genes (*OsRBOHA–OsRBOHI*) were identified in the rice genome (Wong et al., 2007). Rice *RBOH* genes exhibited unique patterns of expression changes in response to various environmental stresses (Wang et al., 2013). A small GTPase Rac in rice (OsRac1) was identified as a positive regulator of OsRBOHB involved in pathogen defense (Wong et al., 2007). A direct interaction between OsRac1 and the N-terminal extension of OsRBOHB may be required for NADPH oxidase activity modulated by the cytosolic Ca^2+^ concentration in plants (Wong et al., 2007). Further mutation analyses of the regulatory domains of OsRBOHB indicated that not only the EF-hand motif but also the upstream N-terminal region was essential to Ca^2+^-dependent but not phosphorylation-dependent activation (Takahashi et al., 2012). In addition, Liu et al. (2012) found that phosphatidylinositol 3-kinase (PI3K) regulated NADPH oxidase activity by modulating the recruitment of Rac1 to plasma membrane. Rice histidine kinase OsHK3 showed to regulate the expression of NADPH oxidase genes and the production of H_2_O_2_ in ABA signaling (Wen et al., 2015). In potato, two CDPKs, StCDPK4 and StCDPK5, were found to induce the phosphorylation of StRBOHB and regulated the oxidative burst during pathogen defense (Kobayashi et al., 2007). In tobacco, NbRBOHA and NbRBOHB are in charge of the generation of ROS during the defense response (Yoshioka et al., 2003). Further study indicated that mitogen-activated protein kinase (MAPK) cascades MEK2-SIPK/NTF4 and MEK1-NTF6 were involved in the NbRBOHB-dependent oxidative burst in response to pathogen signals (Asai et al., 2008). Two tomato RBOH genes, *SlRBOHB* (*SlWfi1*) and *SlRBOHG* (*SlRBOH1*), have turned out to participate in wounding response and development (Sagi et al., 2004). Other studies revealed that SlRBOHG (SlRBOH1) is vital for brassinosteroid (BR)-induced H_2_O_2_ production, ABA accumulation, stomatal closure/opening and oxidative stress tolerance (Xia et al., 2014; Zhou et al., 2014a), while SlRBOHB was found to positively regulate the defense response against *B. cinerea*, the flg22-induced immune response and drought stress response (Li et al., 2015). Lin et al. (2009) observed that the activity of NADPH oxidase is regulated by H_2_O_2_ and ZmMPK5 in maize. Zhu et al. (2013b) identified a BR induced microtubule-associated protein, ZmMAP65-1a, interacts with a MAPK and functions in H_2_O_2_ self-propagation by regulating the expression of NADPH oxidase genes in BR signaling in maize.

## Regulation Of Antioxidative System In Crop Plants

Plant antioxidative system consists of numerous enzymatic and non-enzymatic antioxidative components that work together with ROS-generating pathway to maintain ROS homeostasis. Several studies showed important roles of antioxidative components in ROS homeostasis in crop plants. The rice (*japonica*) genome has eight genes that encode putative SODs, including two cytosolic copper-zinc SODs (*cCuZn-SOD1* and *cCuZn-SOD2*), one putative CuZn-SOD-like *(CuZn-SOD-L*), one plastidic SOD (*pCuZn-SOD*), two iron SODs (*Fe-SOD2* and *Fe-SOD3*), and one manganese SOD (*Mn-SOD1*) (Nath et al., 2014). Transgenic rice plants overexpressing *Mn-SOD1* showed less mitochondrial O_2_^•-^ under stress and reduced the stress induction of *OsAOX1a/b* specifically (Li et al., 2013). There are eight APX genes in rice, including two cytosolic APXs (*OsAPX1* and *OsAPX2*), two peroxisomal APXs (*OsAPX3* and *OsAPX4*), two mitochondrial APXs (*OsAPX5* and *OsAPX6*) and two chloroplastic APXs (*OsAPX7* and *OsAP*X8) (Teixeira et al., 2004, 2006). Two cytosolic APXs, OsAPX1 and OsAPX2, have crucial roles in abiotic stress resistance in rice (Sato et al., 2011; Zhang et al., 2013). Interestingly, rice mutants double silenced for cytosolic APXs (APX1/2s) exhibit significant changes in the redox status indicated by higher H_2_O_2_ levels and increased glutathione and ascorbate redox states, triggering alterations in the ROS signaling networks and making the mutants able to cope with abiotic stress similar to non-transformed plants (Bonifacio et al., 2011). Some of the ROS-scavenging enzymes, such as GST (Dixon and Edwards, 2010), TRX, and GRX (Meyer et al., 2012), have evolved into large multigene families with varied functions that cope with a variety of adverse environmental conditions. Recent mutational and transgenetic plants analyses revealed special member of multigene enzyme family as a key player in ROS homeostasis regulation in crop plants. *OsTRXh1*, encodes h-type TRX in rice, regulates the redox state of the apoplast and participates in plant development and stress responses (Zhang et al., 2011). OsTRXh1 protein possesses reduction activity and secreted into the extracellular space. Overexpression of *OsTRXh1* produce less H_2_O_2_ under salt stress, reduce the expression of the salt-responsive genes, lead to a salt-sensitive phenotype in rice. In another study, Perez-Ruiz et al. (2006) reported that rice NADPH thioredoxin reductase (NTRC) utilizes NADPH to reduce the chloroplast 2-Cys PRX BAS1, thus protects chloroplast against oxidative damage by reducing H_2_O_2_.

The involvement of ROS in signal transduction implies that there must be coordinated function of regulation networks to maintain ROS at non-toxic levels in a delicate balancing act between ROS production and ROS-scavenging pathways, and to regulate ROS responses and subsequent downstream processes (Mittler et al., 2004). Numerous studies from different plant species observed that the generation of ROS and activity of various antioxidant enzymes increased during abiotic stresses (Damanik et al., 2010; Selote and Khanna-Chopra, 2010; Tang et al., 2010; Turan and Ekmekci, 2011). There is an increasing body of literature concerning the mechanisms by which regulation of antioxidative system response to abiotic stresses in crops. Intrinsic to this regulation is ROS production and signaling that integrated with the action of hormone and small molecules.

The plant hormone ABA is the key regulator of abiotic stress resistance in plants, and regulates large number of stress-responsive genes by a complex regulatory network so as to confer tolerance to the environmental stresses (Cutler et al., 2010; Raghavendra et al., 2010). ABA-induced stress tolerance is partly linked with the activation of antioxidant defense systems, including enzymatic and non-enzymatic constituents, which protects plant cells against oxidative damage (Huang et al., 2012; Zhang et al., 2012a, 2014). Water stress-induced ABA accumulation and exogenous ABA treatment triggers the increased generation of ROS, then leads to the activation of the antioxidant system in crops (Jiang and Zhang, 2002a,b; Ye et al., 2011). Small molecules, such as Ca^2+^, calmodulin (CaM), NO and ROS have been demonstrated to play vital roles in ABA-induced antioxidant defense (Jiang and Zhang, 2003; Hu et al., 2007). In rice, a Ca^2+^/CaM-dependent protein kinase (CCaMK), OsDMI3, is necessary for ABA-induced increases in the expression and the activities of SOD and CAT. ABA-induced H_2_O_2_ production activates OsDMI3, and the activation of OsDMI3 also enhances H_2_O_2_ production by increasing the expression of NADPH oxidase genes (Shi et al., 2012). Further study indicated that OsDMI3 functions upstream of OsMPK1, to regulate the activities of antioxidant enzymes and the production of H_2_O_2_ in rice (Shi et al., 2014). Recent study provides evidence to show that rice histidine kinase OsHK3 functions upstream of OsDMI3 and OsMPK1, and is necessary for ABA-induced antioxidant defense (Wen et al., 2015). Zhang et al. (2012a) reported that C2H2-type ZFP, ZFP182, is involved in ABA-induced antioxidant defense. Another C2H2-type ZFP, ZFP36, is also necessary for ABA-induced antioxidant defense (Zhang et al., 2014). Moreover, ABA-induced H_2_O_2_ production and ABA-induced activation of OsMPKs promote the expression of *ZFP36*, and *ZFP36* also up-regulates the expression of NADPH oxidase and MAPK genes and the production of H_2_O_2_ in ABA signaling (Zhang et al., 2014). In maize, ABA and H_2_O_2_ increased the expression and the activity of ZmMPK5, which is required for ABA-induced antioxidant defense. The activation of ZmMPK5 also enhances the H_2_O_2_ production by increasing the expression and the activity of NADPH oxidase, thus there is a positive feedback loop involving NADPH oxidase, H_2_O_2_, and ZmMPK5 in ABA signaling (Zhang et al., 2006; Hu et al., 2007; Ding et al., 2009; Lin et al., 2009). Subsequent experiments confirmed that ABA-induced H_2_O_2_ production mediates NO generation in maize leaves, which, in turn, activates MAPK and increases the expression and the activities of antioxidant enzymes in ABA signaling (Zhang et al., 2007). Moreover, a maize CDPK gene, *ZmCPK11*, acts upstream of ZmMPK5, is essential for ABA-induced up-regulation of the expression and activities of SOD and APX, and the production of H_2_O_2_ in maize leaves (Ding et al., 2013). Hu et al. (2007) found that Ca^2+^-CaM is required for ABA-induced antioxidant defense and functions both upstream and downstream of H_2_O_2_ production in leaves of maize plants. Afterward, Ca^2+^/CaM-dependent protein kinase, ZmCCaMK, was reported to be essential for ABA-induced antioxidant defense, and H_2_O_2_-induced NO production is involved in the activation of ZmCCaMK in ABA signaling (Ma et al., 2012).

Brassinosteroids are a group of steroid hormones and important for a broad spectrum of plant growth and development processes, as well as responses to biotic and abiotic stresses (Bajguz and Hayat, 2009; Divi and Krishna, 2009; Yang et al., 2011; Zhu et al., 2013a). Numerous studies have shown that BR can activate antioxidant defense systems to improve stress tolerance in crops (Özdemir et al., 2004; Xia et al., 2009). Zhang et al. (2010) reported that ZmMPK5 is required for NADPH oxidase-dependent self-propagation of ROS in BR-induced antioxidant defense systems in maize. Further study founded that a 65 kDa microtubule-associated protein (MAP65), ZmMAP65-1a, directly phosphorylated by ZmMPK5, is required for BR-induced antioxidant defense (Zhu et al., 2013b). Recently, Ca^2+^ and maize CCaMK gene, *ZmCCaMK*, was demonstrated to be required for BR-induced antioxidant defense (Yan et al., 2015).

## Genes Involved In Ros Regulation And Abiotic Stress Tolerance In Crops

**FIGURE 1:**
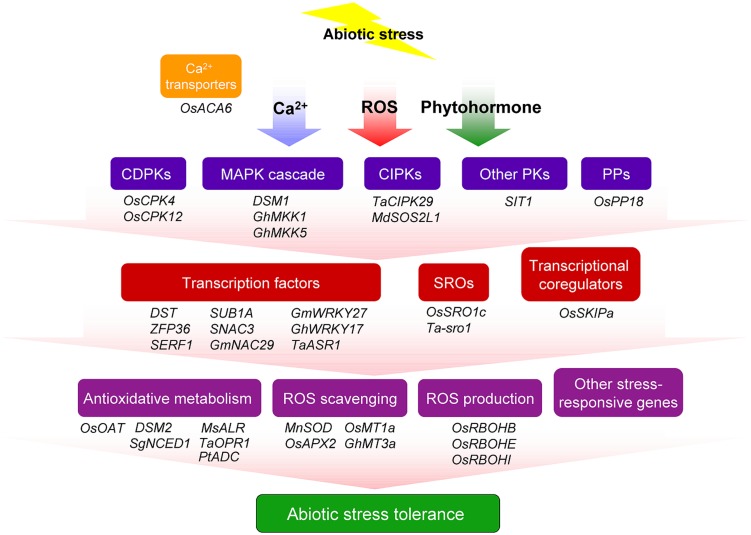
**Overview of major genes that involved in abiotic stress resistance through ROS regulation in crop plants.** Plant cells perceive abiotic stress signals and transduce them through various signaling pathways including secondary signaling molecules, plant hormones, and transcriptional regulators. The regulation of gene expression by different transcription regulators results in the induction of various defense pathways, such as, reactive oxygen species (ROS) scavenging and antioxidative metabolism. Transcription regulators also mediate ROS producing systems and activate the expression of stress-responsive gene so as to confer tolerance to the environmental stresses. CDPK, calcium-dependent protein kinase; CIPK, calcineurin B-like protein-interacting protein kinase; MAPK, mitogen-activated protein kinase; PK, protein kinase; PP, protein phosphatase; SRO, similar to RCD one.

To cope with abiotic stress, plants have evolved multiple and interconnected signaling pathways to regulate different sets of stress-responsive genes for producing various classes of proteins, such as protein kinases, transcriptional factors, enzymes, molecular chaperones, and other functional proteins, resulting in diverse physiological and metabolic response so as to confer tolerance to the environmental stresses. Hundreds or even 1000s of genes that regulate stress responses have been identified in crop plants by diverse functional genomics approaches (Hu and Xiong, 2014). In parallel to this, the functions of numerous stress-responsive genes involved in ROS homeostasis regulation and abiotic stress resistance have been characterized in transgenic plants (**Figure 1**; **Table 1**).

**Table 1:** Representative genes that involved in abiotic stress resistance in major crops through ROS regulation.

Functional category	Genes	Protein function	Origin	Transformation receptor	ROS regulation	Abiotic stress resistance	Reference
**Protein kinase**							
MAPKs	*GhMKK1*	MAPKK	*G. hirsutum*	*N. benthamiana*	ROS scavenging	Drought and salt stress	Lu et al., 2013
	*DSM1*	MAPKKK	*O. sativa*	*O. sativa*	ROS scavenging	Drought stress	Ning et al., 2010
CDPK	*OsCPK4*	calcium-dependent protein kinase	*O. sativa*	*O. sativa*	ROS scavenging	Drought and salt stress	Campo et al., 2014
	*OsCPK12*	calcium-dependent protein kinase	*O. sativa*	*O. sativa*	ROS production and scavenging	Salt stress	Asano et al., 2012
CIPK	*TaCIPK29*	CBL-interacting protein kinase	*T. aestivum*	*N. benthamiana*	ROS scavenging	salt stress	Deng et al., 2013
	*MdSOS2L1*	CBL-interacting protein kinase	*Malus x domestica*	*Malus x domestica*; *S. lycopersicum*	ROS scavenging; antioxidative metabolism	Salt stress	Hu et al., 2015
Other kinase	*SIT1*	Lectin receptor-like kinase	*O. sativa*	*O. sativa*	ROS production	Salt stress	Li et al., 2014
Protein phosphatase	*OsPP18*	Protein phosphatase 2C	*O. sativa*	*O. sativa*	ROS scavenging	Drought and oxidative stress	You et al., 2014
**Transcription factors**							
Zinc finger	*DST*	C2H2 zinc finger	*O. sativa*	*O. sativa*	ROS scavenging	Drought and salt stress	Huang et al., 2009
	*ZFP36*	C2H2 zinc finger	*O. sativa*	*O. sativa*	ABA-induced antioxidant defense	Drought and oxidative stress	Zhang et al., 2014
	*OsTZF1*	CCCH zinc finger	*O. sativa*	*O. sativa*	ROS scavenging	Drought, salt and oxidative stress	Jan et al., 2013
AP2/ERF	*SERF1*	ERF	*O*. *sativa*	*O. sativa*	ROS signaling	Salt stress	Schmidt et al., 2013
	*SUB1A*	ERF	*O*. *sativa*	*O. sativa*	ROS scavenging	Drought, submerge and oxidative stress	Fukao et al., 2011
	*JERF3*	ERF	*S*. *lycopersicum*	*N. benthamiana*	ROS scavenging	Drought, salt and freezing stress	Wu et al., 2008
WRKY	*GmWRKY27*	WRKY	*G*. *max*	*G. max*	ROS production	Drought and salt stress	Wang et al., 2015
	*GhWRKY17*	WRKY	*G*. *hirsutum*	*N. benthamiana*	ROS scavenging	Drought and salt stress	Yan et al., 2014
NAC	*GmNAC29*	NAC	*G*. *max*	*G. max*	ROS production	Drought and salt stress	Wang et al., 2015
	*SNAC3*	NAC	*O. sativa*	*O. sativa*	ROS scavenging	Drought, heat and oxidative stress	Fang et al., 2015
Other TF	*TaASR1*	ASR	*T*. *aestivum*	*N. benthamiana*	ROS scavenging	Drought and oxidative stress	Hu et al., 2013
**Other nuclear proteins**							
SRO protein	*OsSRO1c*	SRO	*O*. *sativa*	*O. sativa*	ROS scavenging	Drought and oxidative stress	You et al., 2013
	*Ta-sro1*	SRO	*T*. *aestivum*	*T. aestivum; A. thaliana*	ROS production and scavenging	Osmotic, salt and oxidative stress	Liu et al., 2014
Other	*OsSKIPa*	Ski-interaction protein	*O*. *sativa*	*O. sativa*	ROS scavenging	Drought stress	Hou et al., 2009
ABA metabolism	*DSM2*	Carotene hydroxylase	*O*. *sativa*	*O. sativa*	antioxidative metabolism	Drought and oxidative stress	Du et al., 2010
	*SgNCED1*	9-*cis*-epoxycarotenoid	*S*. *guianensis*	*N. benthamiana*	ABA-induced antioxidant defense	Drought and salt stress	Zhang et al., 2009
ROS scavenging	*MnSOD*	MnSOD	*N*. *plumbaginifolia*	*M. sativa*	ROS scavenging	Drought stress	McKersie et al., 1996
	*OsAPX2*	APX	*O*. *sativa*	*O. sativa*	ROS scavenging	Drought, salt and cold stresses	Zhang et al., 2013
Detoxification proteins	*MsALR*	NADPH-dependent aldose/aldehyde reductase	*M*. *sativa*	*N. benthamiana*	antioxidative metabolism	Drought and oxidative stress	Oberschall et al., 2000
	*OsMT1a*	type 1 metallothionein	*O*. *sativa*	*O. sativa*	ROS scavenging	Drought stress	Yang et al., 2009
	*GhMT3a*	Type 3 metallothionein	*G*. *hirsutum*	*N. benthamiana*	ROS scavenging	Drought, salt and cold stresses	Xue et al., 2009
Calcium transporters	*OsACA6*	type IIB Ca^2+^ATPase	*O*. *sativa*	*N. benthamiana*	ROS scavenging	Drought and salt stress	Huda et al., 2013
Polyamines metabolism	*PtADC*	Arginine decarboxylase	*P*. *trifoliata*	*N. benthamiana*; *L. esculentum*	ROS scavenging	Drought stress	Wang et al., 2011
Amino acid metabolism	*OsOAT*	Ornithine δ-aminotransferase	*O*. *sativa*	*O. sativa*	antioxidative metabolism; ROS scavenging	Drought and oxidative stress	You et al., 2012
Helicase	*OsSUV3*	NTP-dependent RNA/DNA helicase	*O*. *sativa*	*O. sativa*	ROS scavenging	Drought and salt stress	Tuteja et al., 2013
Unknown function	*TaOPR1*	12-oxo-phytodienoic acid reductases	*T*. *aestivum*	*T. aestivum; A. thaliana*	ABA-induced antioxidant defense	Salt and oxidative stress	Dong et al., 2013


### Protein Kinases and Phosphatases

Mitogen-activated protein kinase cascades are involved in diverse processes from plant growth and development to stress responses. MAPK cascades also play crucial roles in ROS signaling, and several studies in *Arabidopsis* have shown that ROS are not only the trigger, but also the consequence of activation of MAPK signaling (Kovtun et al., 2000; Pitzschke and Hirt, 2006; Pitzschke et al., 2009). However, few MAPK cascades components have been functionally characterized in crops. Two MAPK kinases (MAPKKs), GhMKK1 and GhMKK5 have been characterized to be involved in stress resistance and ROS homeostasis in cotton (Zhang et al., 2012b; Lu et al., 2013). Overexpression of *GhMKK1* in tobacco improved its tolerance to salt and drought stresses, exhibited an enhanced ROS scavenging capability and significantly elevated activities of antioxidant enzymes (Lu et al., 2013). Whereas, overexpression of another cotton MAPKK gene, *GhMKK5*, in tobacco reduced their tolerance to salt and drought stresses. *GhMKK5*-overexpressing plants showed significantly up-regulated expression of ROS-related and cell death marker genes, and resulted in excessive accumulation of H_2_O_2_ and hypersensitive response (HR)-like cell death (Zhang et al., 2012b). In another study, a drought-hypersensitive mutant (drought-hypersensitive mutant1 [*dsm1*]) of a putative MAPK kinase kinase gene has been identified in rice (Ning et al., 2010). The *dsm1* mutant was sensitive to oxidative stress with down-regulated expression of two peroxidase (POD) genes and reduced POD activity.

Calcium-dependent protein kinase proteins regulate the downstream components in calcium signaling pathways. A rice CDPK gene, *OsCPK12*, enhances tolerance to salt stress by reducing the accumulation of ROS (Asano et al., 2012). Expression of genes encoding ROS-scavenging enzymes (*OsAPx2* and *OsAPx8*) were up-regulated, whereas the NADPH oxidase gene (*OsRBOHI*) was down-regulated in *OsCPK12*-overexpressing plants compared with wild type plants. Conversely, the *oscpk12* mutant and RNAi plants were more sensitive to high salinity and accumulated more H_2_O_2_ than wild type plants (Asano et al., 2012). Overexpression of another CDPK gene, *OsCPK4*, results in increased tolerance to salt and drought stresses in rice plants. Transgenic plants exhibited higher expression of numerous genes involved in lipid metabolism and protection against oxidative stress, therefore, reduced levels of membrane lipid peroxidation under stress conditions (Campo et al., 2014).

Calcium-dependent protein kinase proteins also have been found to be responsive to abiotic stress via ROS regulation. Overexpression of wheat CIPK gene *TaCIPK29* in tobacco resulted in increased salt tolerance. Transgenic tobacco seedlings maintained high K^+^/Na^+^ ratios and Ca^2+^ content by up-regulating the expression of some transporter genes, and also reduced ROS accumulations by increasing the expression and activities of ROS-scavenging enzymes under salt stress (Deng et al., 2013). Overexpression of *MdSOS2L1*, a CIPK gene from apple, also conferred salt tolerance in apple and tomato (Hu et al., 2015). Molecular analysis and functional characterization of MdSOS2L1 exhibited that it increases the ROS scavenging-enzymes and antioxidant metabolites such as procyanidin and malate, leading to enhanced salt tolerance in apple and tomato (Hu et al., 2015). A rice lectin receptor-like kinase, salt intolerance 1 (SIT1) was demonstrated mediates salt sensitivity by regulating ROS and ethylene homeostasis and signaling (Li et al., 2014). SIT1 phosphorylates MPK3 and 6, and their activation by salt requires SIT1. SIT1 promotes accumulation of ROS, leading to plant death under salt stress, which occurred in an MPK3/6- and ethylene signaling-dependent manner (Li et al., 2014).

The dephosphorylation mediated by protein phosphatase is an important event in the signal transduction process that regulates various cellular activities. A rice protein phosphatase 2C (PP2C) gene, *OsPP18*, was identified as a SNAC1-regulated downstream gene (You et al., 2014). The *ospp18* mutant exhibited sensitive to drought and oxidative stress with reduced activities of ROS-scavenging enzymes. The ABA-induced expression of ABA-responsive genes has not been disrupted in *ospp18* mutant, indicating *OsPP18* mediates drought stress resistance by regulating ROS homeostasis through ABA-independent pathways (You et al., 2014).

### Transcriptional factors

Transcriptional factors (TFs) are one of the important regulatory proteins involved in abiotic stress responses. They play essential roles downstream of stress signaling cascades, which could alter the expression of a subset of stress-responsive genes simultaneously and enhance tolerance to environmental stress in plants. Members of AP2/ERF (APETALA2/ethylene response factor), zinc finger, WRKY, bZIP (basic leucine zipper), and NAC (NAM, ATAF, and CUC) families have been characterized with roles in the regulation of plant abiotic stress responses (Yamaguchi-Shinozaki and Shinozaki, 2006; Ariel et al., 2007; Ciftci-Yilmaz and Mittler, 2008; Fang et al., 2008), and some of them have been demonstrated to be involved in ROS homeostasis regulation and abiotic stress resistance in crops.

Proteins containing zinc finger domain(s) were widely reported to be key players in the regulation of ROS-related defense genes in *Arabidopsis* and other species. For example, the expression of some zinc finger genes in *Arabidopsis*, *ZAT7*, *ZAT10* and *ZAT12*, is intensely up-regulated by oxidative stress in AtAPX1 knockout plants (Miller et al., 2008). Subsequent experiments showed that these zinc finger proteins were involved in ROS regulation and multiple abiotic stresses tolerance (Davletova et al., 2005; Mittler et al., 2006; Ciftci-Yilmaz et al., 2007). The zinc finger proteins are divided into several types, such as C2H2, C2C2, C2HC, CCCH and C3HC4, based on the number and the location of characteristic residues (Ciftci-Yilmaz and Mittler, 2008). The signaling pathways participating in stomatal movement were well studied in the model plant *Arabidopsis*, but were largely unknown in crops. Huang et al. (2009) identified a drought and salt tolerance (*dst*) mutant, and the DST was cloned by the map-based cloning. DST encoded a C2H2-type zinc finger transcription factor that negatively regulated stomatal closure by direct regulation of genes related to H_2_O_2_ homeostasis, which identified a novel signaling pathway of DST-mediated H_2_O_2_-induced stomatal closure (Huang et al., 2009). Loss of DST function increased the accumulation of H_2_O_2_ in guard cell, accordingly, resulted in increased stomatal closure and enhanced drought and salt tolerance in rice. Other two C2H2-type zinc finger proteins, ZFP36 and ZFP179, also play circle role in ROS homeostasis regulation and abiotic stress resistance in rice. *ZFP179* encodes a salt-responsive zinc finger protein with two C2H2-type zinc finger motifs (Sun et al., 2010). The *ZFP179* transgenic rice plants increased ROS-scavenging ability and expression levels of stress-related genes, and exhibited significantly enhanced tolerance to salt and oxidative stress (Sun et al., 2010). *ZFP36* is an ABA and H_2_O_2_-responsive C2H2-type zinc finger protein gene, and plays a important role in ABA-induced antioxidant defense and the tolerance of rice to drought and oxidative stresses (Zhang et al., 2014). Moreover, ZFP36 is a major player in the regulation of the cross-talk involving NADPH oxidase, H_2_O_2_, and MAPK in ABA signaling (Zhang et al., 2014). OsTZF1, a CCCH-tandem zinc finger protein, was identified as a negative regulator of leaf senescence in rice under stress conditions (Jan et al., 2013). Meanwhile, OsTZF1 confers tolerance to oxidative stress in rice by enhancing the expression of redox homeostasis genes and ROS-scavenging enzymes (Jan et al., 2013). A cotton CCCH-type tandem zinc finger gene, *GhTZF1*, also serves as a key player in modulating drought stress resistance and subsequent leaf senescence by mediating ROS homeostasis (Zhou et al., 2014b).

Members of AP2/ERF (APETALA2/ethylene response factor) transcription factor family, including DREB/CBF transcription factors, are especially important as they regulate genes involved in multiple abiotic stress responses (Mizoi et al., 2012). During the initial phase of abiotic stresses, elevated ROS levels might act as a vital acclimation signal. But the key regulatory components of ROS-mediated abiotic stress response signaling are largely unknown. Rice salt- and H_2_O_2_-responsive ERF transcription factor, SERF1, has a critical role in regulating H_2_O_2_-mediated molecular signaling cascade during the initial response to salinity in rice (Schmidt et al., 2013). SERF1 regulates the expression of H_2_O_2_-responsive genes involved in salt stress responses in roots. SERF1 is also a phosphorylation target of a salt-responsive MAPK (MAPK5), and activation the expression of salt-responsive MAPK cascade genes (*MAPK5* and *MAPKKK6*), well established salt-responsive TF genes (*ZFP179* and *DREB2A*), and itself through direct interaction with the corresponding promoters in plants (Schmidt et al., 2013). The authors proposed that SERF1 is essential for the propagation of the initial ROS signal to mediate salt tolerance. SUB1A, an ERF transcription factor found in limited rice accessions, limits ethylene production and gibberellin responsiveness during submergence, economizing carbohydrate reserves and significantly prolonging endurance (Fukao and Xiong, 2013). After floodwaters subside, submerged plants encounter re-exposure to atmospheric oxygen, leading to postanoxic injury and severe leaf desiccation (Setter et al., 2010; Fukao and Xiong, 2013). SUB1A also positively affects postsubmergence responses by restrained accumulation of ROS in aerial tissue during desubmergence (Fukao et al., 2011). Consistently, SUB1A promptes the expression of ROS scavenging enzyme genes, resulting in enhanced tolerance to oxidative stress. On the other hand, SUB1A improves survival of rapid dehydration following desubmergence and water deficit during drought by increasing ABA responses, and activating stress-inducible gene expression (Fukao et al., 2011). A jasmonate and ethylene-responsive ERF gene, JERF3, was isolated from tomato and involved in a ROS-mediated regulatory module in transcriptional networks that govern plant response to stress (Wu et al., 2008). JERF3 modulates the expression of genes involved in osmotic and oxidative stresses responses by binding to the osmotic- and oxidative-responsive related *cis* elements. The expression of these genes leads to reduce accumulation of ROS, resulting in enhanced abiotic stress tolerance in tobacco (Wu et al., 2008).

The WRKY family proteins have one or two conserved WRKY domains comprising a highly conserved WRKYGQK heptapeptide at the N-terminus and a zinc-finger-like motif at the C-terminus (Eulgem et al., 2000). The conserved WRKY domain plays important roles in various physiological processes by binding to the W-box in the promoter regions of target genes (Ulker and Somssich, 2004; Rushton et al., 2010). Wang et al. (2015) reported a multiple stress-responsive WRKY gene, *GmWRKY27*, reduces ROS level and enhances salt and drought tolerance in transgenic soybean hairy roots. GmWRKY27 interacts with GmMYB174, which, in turn, acts in concert to reduce promoter activity and gene expression of *GmNAC29* (Wang et al., 2015). Further experiments showed that GmNAC29 is a negative factor of stress tolerance for enhancing the ROS production under abiotic stress by directly activating the expression of the gene encoding ROS production enzyme. In another study, overexpression of cotton WRKY gene, *GhWRKY17*, reduced transgenic tobacco plants tolerance to drought and salt stress. Subsequent experiments showed that GhWRKY17 involved in stress responses by regulating ABA signaling and cellular levels of ROS (Yan et al., 2014). Sun et al. (2015) isolated a WRKY gene, *BdWRKY36*, from *B. distachyon*, and found it functions as a positive regulator of drought stress response by controlling ROS homeostasis and regulating transcription of stress-related genes.

Members of other TF families also functioned in abiotic stress response through ROS regulation. ASR proteins are plant-specific TFs and considered to be important regulators of plant response to various stresses. Wheat ASR gene, *TaASR1*, a positive regulator of plant tolerance to drought/osmotic stress, is involved in the modulation of ROS homeostasis by activating antioxidant system and transcription of stress-responsive genes (Hu et al., 2013). Soybean NAC TF, GmNAC2, was identified as a negative regulator during abiotic stress, and participates in ROS signaling pathways through modulation of the expression of genes related to ROS-scavenging (Jin et al., 2013). Ramegowda et al. (2012) isolated a stress-responsive NAC gene, *EcNAC1*, from finger millet (*E*. *coracana*). Transgenic tobacco plants expressing *EcNAC1* increased ROS scavenging activity, up-regulated many stress-responsive genes, and exhibited tolerance to various abiotic stresses and MV-induced oxidative stress (Ramegowda et al., 2012). Recently, a NAC transcription factor gene, *SNAC3*, functions as a positive regulator under high temperature and drought stress, was identified in rice (Fang et al., 2015). SNAC3 enhances the abiotic stresses tolerance by modulating H_2_O_2_ homeostasis state through controlling the expression of ROS-associated enzyme genes (Fang et al., 2015).

In addition to TFs, transcriptional coregulator as well as spliceosome component, OsSKIPa, a rice homolog of human Ski-interacting protein (SKIP), has been studied for effects on drought resistance (Hou et al., 2009). *OsSKIPa*-overexpressing rice exhibited significantly enhanced drought stress tolerance at both the seedling and reproductive stages by increased ROS-scavenging ability and transcript levels of many stress-related genes (Hou et al., 2009).

## Sro Proteins

The SRO (SIMILAR TO RCD ONE) protein family was recently identified as a group of plant-specific proteins, and they are characterized by the plant-specific domain architecture which contains a poly (ADP-ribose) polymerase catalytic (PARP) and a C-terminal RCD1-SRO-TAF4 (RST) domain (Jaspers et al., 2010). In addition to these two domains, some SRO proteins have an N-terminal WWE domain. Our limited knowledge of SRO proteins is mainly from the study in *Arabidopsis* mutant *rcd1* (*radical-induced cell death 1*). *rcd1* exhibits pleiotropic phenotypes related to a wide range of exogenous stimulus responses and developmental processes, including sensitivity to apoplastic ROS and salt stress, resistance to chloroplastic ROS caused by methyl viologen (MV) and UV-B irradiation (Ahlfors et al., 2004; Fujibe et al., 2004; Katiyar-Agarwal et al., 2006). RCD1 interacts with SOS1 and a large number of transcription factors which have been identified or predicted to be involved in both development and stress-related processes (Katiyar-Agarwal et al., 2006; Jaspers et al., 2009). Recent study demonstrated that RCD1 is possibly involved in signaling networks that regulate quantitative changes in gene expression in response to ROS (Brosche et al., 2014).

In rice, an SRO protein, OsSRO1c, was characterized as a direct target of the drought stress-related transcription factor SNAC1 (You et al., 2013). *OsSRO1c* was induced in guard cells by drought stress. Overexpression of *OsSRO1c* resulted in accumulated H_2_O_2_ in guard cells, which, in turn, decreased stomatal aperture and reduced water loss. Further experiments indicated that OsSRO1c has dual roles in drought and oxidative stress tolerance of rice by promoting stomatal closure and H_2_O_2_ accumulation through a novel pathway involving the SNAC1 and DST regulators (You et al., 2013). Recently, an SRO gene was also identified to be crucial for salinity stress resistance by modulating redox homeostasis in wheat (Liu et al., 2014). Ta-*sro1*, the allele of the salinity-tolerant bread wheat cultivar Shanrong No. 3, is derived from the wheat parent allele via point mutation. Unlike *Arabidopsis* SRO proteins, Ta-sro1 has PARP activity. Both the overexpression of *Ta-sro1* in wheat and *Arabidopsis* promotes the accumulation of ROS by regulating ROS-associated enzyme. Ta-sro1 also enhances the activity of AsA-GSH cycle enzymes and GPX cycle enzymes, which regulate ROS content and cellular redox homeostasis (Liu et al., 2014).

### ROS-scavenging or Detoxification Proteins

Reactive oxygen species-scavenging enzymes such as SOD, APX, CAT were properly described its role in ROS-scavenging pathway. The presence of antioxidant enzymes and compounds in almost all cellular compartments suggests the importance of ROS detoxification for protection against various stresses (Mittler et al., 2004). The effect of these ROS-scavenging enzymes in abiotic stress resistance was also investigated in crop plants. Transgenic alfalfa expressing MnSOD cDNA from *Nicotiana plumbaginifolia* improved survival and vigor after exposure to water deficit. Most importantly, transgenic alfalfa showed increased yield and survival rate over three winters in natural field environments (McKersie et al., 1996). A cDNA encoding a cytosolic copper-zinc SOD from the mangrove plant *Avicennia marina* was transformed into rice. The transgenic plants exhibited more tolerant to drought, salinity and oxidative stresses compared with the untransformed control plants (Prashanth et al., 2008). Overexpression of *OsAPX2* increased APX activity and reduced H_2_O_2_ and malondialdehyde (MDA) levels in transgenic plants under stress treatments (Zhang et al., 2013). More importantly, *OsAPX2*-overexpressing plants were more tolerant to drought stress than wild-type plants at the booting stage as indicated a significantly increase in spikelet fertility under abiotic stresses (Zhang et al., 2013). Transgenic rice plants that overexpressing another APX gene, *OsAPX1*, also exhibited increased spikelet fertility under cold stress (Sato et al., 2011).

Accumulation of toxic products from ROS with lipids and proteins significantly contributes to the damage of crop plants under biotic and abiotic stresses. A novel plant NADPH-dependent aldose/aldehyde reductase, which has the reduction activity toward toxic products of lipid peroxidation, was isolated from alfalfa. Tobacco plants overproducing the alfalfa aldose/aldehyde reductase showed lower concentrations of reactive aldehydes (products of lipid peroxidation) and tolerance to oxidative and drought stress (Oberschall et al., 2000).

Metallothioneins (MTs) are a group of low molecular weight proteins with the characteristics of high cysteine (Cys) residue content and metal-binding ability. The presence of several Cys residues in MTs suggests their involvement in the detoxification of ROS or in the maintenance of redox levels. *OsMT1a*, encoding a type 1 MT in rice, was induced by dehydration and Zn^2+^ treatment (Yang et al., 2009). Transgenic rice plants overexpressing *OsMT1a* enhanced antioxidant enzyme activities of CAT, POD and APX, and enhanced tolerance to drought. OsMT1a also regulates the expression of several zinc finger transcription factors by the modulation of Zn^2+^ homeostasis, which leads to enhanced plant stress tolerance (Yang et al., 2009). *GhMT3a* encodes a type 3 plant MT in cotton. Recombinant GhMT3a protein showed an ability to bind metal ions and scavenge ROS *in vitro*. Transgenic tobaccos showed more tolerance to multiple abiotic stresses, and lower H_2_O_2_ levels when compared with wild-type plants (Xue et al., 2009). The *SbMT-2* gene from a halophyte was also involved in maintaining cellular homeostasis by regulating ROS scavenging during stresses and thus improved tolerance to salt and osmotic stress in transgenic tobacco (Chaturvedi et al., 2014).

### ABA Metabolic-related Proteins

Abscisic acid is a key phytohormone that medicates the adaptive responses to abiotic stresses of plants. ABA-induced antioxidant defense has been well documented in plants. ABA biosynthesis and catabolism also involved in antioxidant defense and abiotic stresses. Du et al. (2010) isolated a rice drought-sensitive mutant *dsm2*, impaired in the gene encoding a putative β-carotene hydroxylase. β-carotene hydroxylase is predicted for the biosynthesis of zeaxanthin, a carotenoid precursor of ABA. Under drought stress, *dsm2* mutants had reduced zeaxanthin and ABA, lower Fv/Fm and non-photochemical quenching (NPQ) capacity than the wild type. Overexpression of *DSM2* in rice increases the xanthophylls and NPQ capacity, stress-related ABA-responsive genes expression, and resulted in enhancing resistance to drought and oxidative stresses (Du et al., 2010). *OsABA8ox3*, encoding ABA 8′-hydroxylase involved in ABA catabolism, is also a key gene regulating ABA accumulation and anti-oxidative stress capability under drought stress (Nguyen et al., 2015). *OsABA8ox3* RNAi plants exhibited significant improvement in drought stress tolerance. Consistent with this, *OsABA8ox3* RNAi plants showed increased SOD and CAT activities and reduced MDA levels during dehydration treatment. In another study, overexpression of the 9-*cis*-epoxycarotenoid dioxygenase gene from *Stylosanthes guianensis* (*SgNCED1*) in the transgenic tobacco increased ABA content and tolerance to drought and salt stresses (Zhang et al., 2009). Moreover, enhanced abiotic stresses tolerance in transgenic plants is associated with ABA-induced production of H_2_O_2_ and NO, which, in turn, activate the expression and activities of ROS-scavenging enzymes (Zhang et al., 2009).

### Calcium Transporters and Calcium-binding Proteins

Calcium (Ca^2+^) regulates numerous signaling pathways involved in growth, development and stress tolerance. The influx of Ca^2+^ into the cytosol is countered by pumping Ca^2+^ out from the cytosol to restore the basal cytosolic level, and this may be achieved either by P-type Ca^2+^ATPases or antiporters. Huda et al. (2013) report the isolation and characterization of *OsACA6*, which encodes a member of the type IIB Ca^2+^ATPase family from rice. Overexpression of *OsACA6* confers tolerance to salinity and drought stresses in tobacco, which was correlated with reduced accumulation of ROS and enhanced the expression of stress-responsive genes in plants (Huda et al., 2013). In addition, overexpression of *OsACA6* confers Cd^2+^ stress tolerance in transgenic lines by maintaining cellular ion homeostasis and modulating ROS-scavenging pathway (Shukla et al., 2014). Annexins are calcium-dependent, phospholipid-binding proteins with suggested functions in response to environmental stresses and signaling during plant growth and development. OsANN1, a member of the annexin protein family in rice, has ATPase activity, the ability to bind Ca^2+^, and the ability to bind phospholipids in a Ca^2+^-dependent manner. OsANN1 confers abiotic stress tolerance by modulating antioxidant accumulation and interacting with OsCDPK24 (Qiao et al., 2015).

### Other Functional Proteins

Polyamines are low molecular weight aliphatic amines found in all living cells. Because of their cationic nature at physiological pH, PAs have strong binding capacity to negatively charged molecules (DNA, RNA, and protein), thus stabilizing their structure (Alcazar et al., 2010). The PAs biosynthetic pathway has been thoroughly investigated in many organisms, and arginine decarboxylase (ADC) plays a predominant role in the accumulation of PAs under stresses (Capell et al., 2004; Alcazar et al., 2010). Wang et al. (2011) isolated an arginine decarboxylase gene (*PtADC*) from *Poncirus trifoliata*. The transgenic tobacco and tomato plants elevated endogenous PAs level, accumulated less ROS and showed an improvement in drought tolerance. Jang et al. (2012) identified a highly oxidative stress-resistant T-DNA mutant line carried an insertion in *OsLDC-like 1* in rice. The mutant produced much higher levels of PAs compared to the wild type plants. Based on their results, the authors suggested that PAs mediate tolerance to abiotic stresses through their ability to decrease ROS generation and enhance ROS degradation.

The 12-oxo-phytodienoic acid reductases (OPRs) are classified into two subgroups, OPRI and OPRII. OPRII proteins are involved in jasmonic acid synthesis, while the function of OPRI is as yet unclear. Dong et al. (2013) characterizated the functions of the wheat OPRI gene *TaOPR1*. Overexpression of *TaOPR1* in wheat and *Arabidopsis* enhanced tolerance to salt stress by regulating of ROS and ABA signaling pathways (Dong et al., 2013).

Helicases are ubiquitous enzymes that catalyze the unwinding of energetically stable duplex DNA or RNA secondary structures, and thereby play an important role in almost all DNA and/or RNA metabolic processes. OsSUV3, an NTP-dependent RNA/DNA helicase in rice, exhibits ATPase, RNA and DNA helicase activities (Tuteja et al., 2013). *OsSUV3* sense transgenic rice plants showed lesser lipid peroxidation and H_2_O_2_ production, along with higher activities of antioxidant enzymes, consequently resulting in increased tolerance to high salinity (Tuteja et al., 2013).

Ornithine *δ*-aminotransferase (*δ*-OAT) is considered to be an enzyme involved in proline and arginine metabolism. *OsOAT*-overexpressing rice plants exhibited significantly increased *δ*-OAT activity and proline levels under normal growth conditions, and enhanced drought, osmotic, and oxidative stress tolerance (You et al., 2012).

## Conclusion And Perspectives

The discovery of the enzymatic activity of SOD 45 years ago (McCord and Fridovich, 1969) ushered in the field of ROS biology. During the last two decades, the major sources and sites of ROS production, and the key antioxidant molecules and enzymes that scavenge ROS have been chartered in plant. However, our current knowledge about ROS homeostasis and signaling remains fragmental. Apoplastic ROS are rapidly produced in plants as a defense response to pathogen attack and abiotic stress. Whereas, in addition to NADPH oxidase, the function and regulation of other apoplastic ROS-associated enzymes, such as class III peroxidases, in stress responses signaling are largely unknown. On the other hand, 100s of genes that encode for ROS-metabolizing enzymes and regulators compose ROS gene network in plants. Thus, more than one enzymatic activity that produces or scavenges ROS exits in certain cellular compartment. How these different enzymes are coordinated within each compartment and between different compartments to adjust a particular ROS at an appropriate level during stresses is an important question needs to be addressed. There is increasing evidence suggesting the vital role of ROS signaling pathway in plant development and stress responses. However, regulatory mechanisms at the biochemical level, the mechanisms of extracellular ROS perception, transduction of ROS-derived signals, and especially the communication and interaction between different subcellular compartments in ROS signaling are still poorly understood. To build comprehensive regulation networks in ROS signaling and responses requires a combination of transcriptomics, proteomics and metabolomics approaches with analysis of mutant as well as protein–protein interactions.

Plants need diverse responses and adjustment of multiple adaptation mechanisms to cope with the multiple stresses exist in nature. Comparison of transcription profiles of rice in response to multiple stresses suggested the central role of ROS homeostasis in different abiotic stresses (Mittal et al., 2012). Therefore, manipulating endogenous ROS levels provides us with an opportunity to improve common defense mechanisms against different stresses to ensure crop plants growth and survival under adverse growing condition. The functions of numerous stress-responsive genes involved in ROS homeostasis regulation and abiotic stress resistance have been characterized in transgenic plants (**Table 1**). As expected, transgenic crop plants harbored these genes enhanced tolerance to multiple abiotic stresses (Wu et al., 2008; Fukao et al., 2011; Lu et al., 2013; Campo et al., 2014). However, few studies have reported the abiotic stress tolerance of transgenic plant at the reproductive or flowering stage based on yield and/or setting rate, and very few of these tests were conducted under field conditions. Additionally, most of the reported ROS-associated genes that involved in abiotic stress just have been demonstrated its role in regulation of expression and/or activity of ROS-scavenging enzymes. Thus, network involving in function of these genes in ROS homeostasis to medicate abiotic stress resistance needs to be fully investigated, and the new components need to be integrated into the signaling pathway. With a long-term goal to improve the abiotic stress resistance of crop plants by the utilizing of ROS regulation pathways, more and more key regulators need to be identified. It is also very important to clarify the mechanisms regulating ROS signaling pathways and their interplay during abiotic stresses. This can finally help to incorporate multiple necessary ROS-associated genes into the genetic backgrounds of elite cultivars or hybrids to enhance their abiotic stress resistance under real agricultural field conditions.

## Conflict of Interest Statement

The authors declare that the research was conducted in the absence of any commercial or financial relationships that could be construed as a potential conflict of interest.

## Abbreviations

ABA: abscisic acid
AOX: alternative oxidases
APX: ascorbate peroxidase
AsA: ascorbic acid
ASR: ABA-, stress-, and ripening-induced
BR: brassinosteroid
CCaMK: calcium/calmodulin-dependent protein kinase
CDPK: calcium-dependent protein kinase
CIPK: calcineurin B-like protein-interacting protein kinase
DHAR: dehydroascorbate reductase
GPX: glutathione peroxidase
GR: glutathione reductase
GRX: glutaredoxin
GSH: reduced glutathione
GST: glutathione S-transferase
MAPK: mitogen-activated protein kinase
MAPKKK: MAPK kinase kinase
MDHAR: monodehydroascorbate reductase
MT: metallothionein
PAs: polyamines
POD: peroxidase
PRX: peroxiredoxin
RBOH: respiratory burst oxidase homolog
RCD: radical-induced cell death
ROS: reactive oxygen species
SOD: superoxide dismutase
SRO: similar to RCD one
TRX: thioredoxin

## References

[B1] Ahlfors R. Lang S. Overmyer K. Jaspers P. Brosche M. Tauriainen A. (2004). *Arabidopsis* RADICAL-INDUCED CELL DEATH1 belongs to the WWE protein-protein interaction domain protein family and modulates abscisic acid, ethylene, and methyl jasmonate responses. *Plant Cell* 16 1925–1937. 10.1105/tpc.021832 15208394 PMC514171

[B2] Alcazar R. Altabella T. Marco F. Bortolotti C. Reymond M. Koncz C. (2010). Polyamines: molecules with regulatory functions in plant abiotic stress tolerance. *Planta* 231 1237–1249. 10.1007/s00425-010-1130-0 20221631

[B3] Apel K. Hirt H. (2004). Reactive oxygen species: metabolism, oxidative stress, and signal transduction. *Annu. Rev. Plant Biol.* 55 373–399. 10.1146/annurev.arplant.55.031903.141701 15377225

[B4] Ariel F. D. Manavella P. A. Dezar C. A. Chan R. L. (2007). The true story of the HD-Zip family. *Trends Plant Sci.* 12 419–426. 10.1016/j.tplants.2007.08.003 17698401

[B5] Asai S. Ohta K. Yoshioka H. (2008). MAPK signaling regulates nitric oxide and NADPH oxidase-dependent oxidative bursts in *Nicotiana benthamiana*. *Plant Cell* 20 1390–1406. 10.1105/tpc.107.055855 18515503 PMC2438462

[B6] Asano T. Hayashi N. Kobayashi M. Aoki N. Miyao A. Mitsuhara I. (2012). A rice calcium-dependent protein kinase OsCPK12 oppositely modulates salt-stress tolerance and blast disease resistance. *Plant J.* 69 26–36. 10.1111/j.1365-313X.2011.04766.x 21883553

[B7] Bajguz A. Hayat S. (2009). Effects of brassinosteroids on the plant responses to environmental stresses. *Plant Physiol. Biochem.* 47 1–8. 10.1016/j.plaphy.2008.10.002 19010688

[B8] Baxter A. Mittler R. Suzuki N. (2014). ROS as key players in plant stress signalling. *J. Exp. Bot.* 65 1229–1240. 10.1093/jxb/ert375 24253197

[B9] Bonifacio A. Martins M. O. Ribeiro C. W. Fontenele A. V. Carvalho F. E. Margis-Pinheiro M. (2011). Role of peroxidases in the compensation of cytosolic ascorbate peroxidase knockdown in rice plants under abiotic stress. *Plant Cell Environ.* 34 1705–1722. 10.1111/j.1365-3040.2011.02366.x 21631533

[B10] Brosche M. Blomster T. Salojarvi J. Cui F. Sipari N. Leppala J. (2014). Transcriptomics and functional genomics of ROS-induced cell death regulation by RADICAL-INDUCED CELL DEATH1. *PLoS Genet.* 10:e1004112 10.1371/journal.pgen.1004112 PMC3923667 24550736

[B11] Campo S. Baldrich P. Messeguer J. Lalanne E. Coca M. San Segundo B. (2014). Overexpression of a calcium-dependent protein kinase confers salt and drought tolerance in rice by preventing membrane lipid peroxidation. *Plant Physiol.* 165 688–704. 10.1104/pp.113.230268 24784760 PMC4044838

[B12] Capell T. Bassie L. Christou P. (2004). Modulation of the polyamine biosynthetic pathway in transgenic rice confers tolerance to drought stress. *Proc. Natl. Acad. Sci. U.S.A.* 101 9909–9914. 10.1073/pnas.0306974101 15197268 PMC470772

[B13] Chaturvedi A. K. Patel M. K. Mishra A. Tiwari V. Jha B. (2014). The SbMT-2 gene from a halophyte confers abiotic stress tolerance and modulates ROS scavenging in transgenic tobacco. *PLoS ONE* 9:e111379 10.1371/journal.pone.0111379 PMC4207811 25340650

[B14] Ciftci-Yilmaz S. Mittler R. (2008). The zinc finger network of plants. *Cell Mol. Life Sci.* 65 1150–1160. 10.1007/s00018-007-7473-4 18193167 PMC11131624

[B15] Ciftci-Yilmaz S. Morsy M. R. Song L. Coutu A. Krizek B. A. Lewis M. W. (2007). The EAR-motif of the Cys2/His2-type zinc finger protein Zat7 plays a key role in the defense response of *Arabidopsis* to salinity stress. *J. Biol. Chem.* 282 9260–9268. 10.1074/jbc.M611093200 17259181

[B16] Cosio C. Dunand C. (2009). Specific functions of individual class III peroxidase genes. *J. Exp. Bot.* 60 391–408. 10.1093/jxb/ern318 19088338

[B17] Couee I. Sulmon C. Gouesbet G. El Amrani A. (2006). Involvement of soluble sugars in reactive oxygen species balance and responses to oxidative stress in plants. *J. Exp. Bot.* 57 449–459. 10.1093/jxb/erj027 16397003

[B18] Cutler S. R. Rodriguez P. L. Finkelstein R. R. Abrams S. R. (2010). Abscisic acid: emergence of a core signaling network. *Annu. Rev. Plant Biol.* 61 651–679. 10.1146/annurev-arplant-042809-112122 20192755

[B19] Damanik R. I. Maziah M. Ismail M. R. Ahmad S. Zain A. (2010). Responses of the antioxidative enzymes in Malaysian rice (*Oryza sativa* L.) cultivars under submergence condition. *Acta Physiol. Plant.* 32 739–747. 10.1007/s11738-009-0456-3

[B20] Davletova S. Schlauch K. Coutu J. Mittler R. (2005). The zinc-finger protein Zat12 plays a central role in reactive oxygen and abiotic stress signaling in *Arabidopsis*. *Plant Physiol.* 139 847–856. 10.1104/pp.105.068254 16183833 PMC1256000

[B21] Deng X. Hu W. Wei S. Zhou S. Zhang F. Han J. (2013). TaCIPK29, a CBL-interacting protein kinase gene from wheat, confers salt stress tolerance in transgenic tobacco. *PLoS ONE* 8:e69881 10.1371/journal.pone.0069881 PMC3726728 23922838

[B22] Dietz K. J. Jacob S. Oelze M. L. Laxa M. Tognetti V. De Miranda S. M. (2006). The function of peroxiredoxins in plant organelle redox metabolism. *J. Exp. Bot.* 57 1697–1709. 10.1093/jxb/erj160 16606633

[B23] Ding H. Zhang A. Wang J. Lu R. Zhang H. Zhang J. (2009). Identity of an ABA-activated 46 kDa mitogen-activated protein kinase from *Zea mays* leaves: partial purification, identification and characterization. *Planta* 230 239–251. 10.1007/s00425-009-0938-y 19424717

[B24] Ding Y. Cao J. Ni L. Zhu Y. Zhang A. Tan M. (2013). ZmCPK11 is involved in abscisic acid-induced antioxidant defence and functions upstream of ZmMPK5 in abscisic acid signalling in maize. *J. Exp. Bot.* 64 871–884. 10.1093/jxb/ers366 23268839 PMC3580805

[B25] Divi U. K. Krishna P. (2009). Brassinosteroid: a biotechnological target for enhancing crop yield and stress tolerance. *N. Biotechnol.* 26 131–136. 10.1016/j.nbt.2009.07.006 19631770

[B26] Dixon D. P. Edwards R. (2010). Glutathione transferases. *Arabidopsis Book* 8 e0131 10.1199/tab.0131 PMC3244946 22303257

[B27] Dong W. Wang M. Xu F. Quan T. Peng K. Xiao L. (2013). Wheat oxophytodienoate reductase gene TaOPR1 confers salinity tolerance via enhancement of abscisic acid signaling and reactive oxygen species scavenging. *Plant Physiol.* 161 1217–1228. 10.1104/pp.112.211854 23321418 PMC3585591

[B28] Du H. Wang N. Cui F. Li X. Xiao J. Xiong L. (2010). Characterization of the beta-carotene hydroxylase gene DSM2 conferring drought and oxidative stress resistance by increasing xanthophylls and abscisic acid synthesis in rice. *Plant Physiol.* 154 1304–1318. 10.1104/pp.110.163741 20852032 PMC2971608

[B29] Duan Z. Q. Bai L. Zhao Z. G. Zhang G. P. Cheng F. M. Jiang L. X. (2009). Drought-stimulated activity of plasma membrane nicotinamide adenine dinucleotide phosphate oxidase and its catalytic properties in rice. *J. Integr. Plant Biol.* 51 1104–1115. 10.1111/j.1744-7909.2009.00879.x 20021558

[B30] Dubiella U. Seybold H. Durian G. Komander E. Lassig R. Witte C. P. (2013). Calcium-dependent protein kinase/NADPH oxidase activation circuit is required for rapid defense signal propagation. *Proc. Natl. Acad. Sci. U.S.A.* 110 8744–8749. 10.1073/pnas.1221294110 23650383 PMC3666735

[B31] Eckardt N. A. Cominelli E. Galbiati M. Tonelli C. (2009). The future of science: food and water for life. *Plant Cell* 21 368–372. 10.1105/tpc.109.066209 19252079 PMC2660623

[B32] Eulgem T. Rushton P. J. Robatzek S. Somssich I. E. (2000). The WRKY superfamily of plant transcription factors. *Trends Plant Sci.* 5 199–206. 10.1016/S1360-1385(00)01600-9 10785665

[B33] Fang Y. Liao K. Du H. Xu Y. Song H. Li X. (2015). A stress-responsive NAC transcription factor SNAC3 confers heat and drought tolerance through modulation of reactive oxygen species in rice. *J. Exp. Bot.* 66:6803 10.1093/jxb/erv386 PMC4623689 26261267

[B34] Fang Y. You J. Xie K. Xie W. Xiong L. (2008). Systematic sequence analysis and identification of tissue-specific or stress-responsive genes of NAC transcription factor family in rice. *Mol. Genet. Genomics* 280 547–563. 10.1007/s00438-008-0386-6 18813954

[B35] Fujibe T. Saji H. Arakawa K. Yabe N. Takeuchi Y. Yamamoto K. T. (2004). A methyl viologen-resistant mutant of *Arabidopsis*, which is allelic to ozone-sensitive rcd1, is tolerant to supplemental ultraviolet-B irradiation. *Plant Physiol.* 134 275–285. 10.1104/pp.103.033480 14657410 PMC316307

[B36] Fukao T. Xiong L. (2013). Genetic mechanisms conferring adaptation to submergence and drought in rice: simple or complex? *Curr. Opin. Plant Biol.* 16 196–204. 10.1016/j.pbi.2013.02.003 23453780

[B37] Fukao T. Yeung E. Bailey-Serres J. (2011). The submergence tolerance regulator SUB1A mediates crosstalk between submergence and drought tolerance in rice. *Plant Cell* 23 412–427. 10.1105/tpc.110.080325 21239643 PMC3051255

[B38] Gill S. S. Tuteja N. (2010). Reactive oxygen species and antioxidant machinery in abiotic stress tolerance in crop plants. *Plant Physiol. Biochem.* 48 909–930. 10.1016/j.plaphy.2010.08.016 20870416

[B39] Hirayama T. Shinozaki K. (2010). Research on plant abiotic stress responses in the post-genome era: past, present and future. *Plant J.* 61 1041–1052. 10.1111/j.1365-313X.2010.04124.x 20409277

[B40] Hou X. Xie K. Yao J. Qi Z. Xiong L. (2009). A homolog of human ski-interacting protein in rice positively regulates cell viability and stress tolerance. *Proc. Natl. Acad. Sci. U.S.A.* 106 6410–6415. 10.1073/pnas.0901940106 19339499 PMC2669339

[B41] Hu D. G. Ma Q. J. Sun C. H. Sun M. H. You C. X. Hao Y. J. (2015). Overexpression of MdSOS2L1, a CIPK protein kinase, increases the antioxidant metabolites to enhance salt tolerance in apple and tomato. *Physiol. Plant* 10.1111/ppl.12354 [Epub ahead of print]. 26096498

[B42] Hu H. Xiong L. (2014). Genetic engineering and breeding of drought-resistant crops. *Annu. Rev. Plant Biol.* 65 715–741. 10.1146/annurev-arplant-050213-040000 24313844

[B43] Hu W. Huang C. Deng X. Zhou S. Chen L. Li Y. (2013). TaASR1, a transcription factor gene in wheat, confers drought stress tolerance in transgenic tobacco. *Plant Cell Environ.* 36 1449–1464. 10.1111/pce.12074 23356734

[B44] Hu X. Jiang M. Zhang J. Zhang A. Lin F. Tan M. (2007). Calcium-calmodulin is required for abscisic acid-induced antioxidant defense and functions both upstream and downstream of H_2_O_2_ production in leaves of maize (*Zea mays*) plants. *New Phytol.* 173 27–38. 10.1111/j.1469-8137.2006.01888.x 17176391

[B45] Huang J. Sun S. Xu D. Lan H. Sun H. Wang Z. (2012). A TFIIIA-type zinc finger protein confers multiple abiotic stress tolerances in transgenic rice (*Oryza sativa* L.). *Plant Mol. Biol.* 80 337–350. 10.1007/s11103-012-9955-5 22930448

[B46] Huang X. Y. Chao D. Y. Gao J. P. Zhu M. Z. Shi M. Lin H. X. (2009). A previously unknown zinc finger protein, DST, regulates drought and salt tolerance in rice via stomatal aperture control. *Genes Dev.* 23 1805–1817. 10.1101/gad.1812409 19651988 PMC2720257

[B47] Huda K. M. Banu M. S. Garg B. Tula S. Tuteja R. Tuteja N. (2013). OsACA6, a P-type IIB Ca2+ ATPase promotes salinity and drought stress tolerance in tobacco by ROS scavenging and enhancing the expression of stress-responsive genes. *Plant J.* 76 997–1015. 10.1111/tpj.12352 24128296

[B48] Jan A. Maruyama K. Todaka D. Kidokoro S. Abo M. Yoshimura E. (2013). OsTZF1, a CCCH-tandem zinc finger protein, confers delayed senescence and stress tolerance in rice by regulating stress-related genes. *Plant Physiol.* 161 1202–1216. 10.1104/pp.112.205385 23296688 PMC3585590

[B49] Jang S. J. Wi S. J. Choi Y. J. An G. Park K. Y. (2012). Increased polyamine biosynthesis enhances stress tolerance by preventing the accumulation of reactive oxygen species: T-DNA mutational analysis of *Oryza sativa* lysine decarboxylase-like protein 1. *Mol. Cells* 34 251–262. 10.1007/s10059-012-0067-5 22965749 PMC3887846

[B50] Jaspers P. Blomster T. Brosche M. Salojarvi J. Ahlfors R. Vainonen J. P. (2009). Unequally redundant RCD1 and SRO1 mediate stress and developmental responses and interact with transcription factors. *Plant J.* 60 268–279. 10.1111/j.1365-313X.2009.03951.x 19548978

[B51] Jaspers P. Overmyer K. Wrzaczek M. Vainonen J. P. Blomster T. Salojarvi J. (2010). The RST and PARP-like domain containing SRO protein family: analysis of protein structure, function and conservation in land plants. *BMC Genomics* 11:170 10.1186/1471-2164-11-170 PMC2848248 20226034

[B52] Jiang M. Zhang J. (2002a). Involvement of plasma-membrane NADPH oxidase in abscisic acid- and water stress-induced antioxidant defense in leaves of maize seedlings. *Planta* 215 1022–1030. 10.1007/s00425-002-0829-y 12355163

[B53] Jiang M. Zhang J. (2002b). Water stress-induced abscisic acid accumulation triggers the increased generation of reactive oxygen species and up-regulates the activities of antioxidant enzymes in maize leaves. *J. Exp. Bot.* 53 2401–2410. 10.1093/jxb/erf090 12432032

[B54] Jiang M. Zhang J. (2003). Cross-talk between calcium and reactive oxygen species originated from NADPH oxidase in abscisic acid-induced antioxidant defence in leaves of maize seedlings. *Plant Cell Environ.* 26 929–939. 10.1046/j.1365-3040.2003.01025.x 12803620

[B55] Jin H. X. Huang F. Cheng H. Song H. N. Yu D. Y. (2013). Overexpression of the GmNAC2 gene, an NAC transcription factor, reduces abiotic stress tolerance in tobacco. *Plant Mol. Biol. Rep.* 31 435–442. 10.1007/s11105-012-0514-7

[B56] Katiyar-Agarwal S. Zhu J. Kim K. Agarwal M. Fu X. Huang A. (2006). The plasma membrane Na+/H+ antiporter SOS1 interacts with RCD1 and functions in oxidative stress tolerance in *Arabidopsis*. *Proc. Natl. Acad. Sci. U.S.A.* 103 18816–18821. 10.1073/pnas.0604711103 17023541 PMC1693745

[B57] Keunen E. Peshev D. Vangronsveld J. Van Den Ende W. Cuypers A. (2013). Plant sugars are crucial players in the oxidative challenge during abiotic stress: extending the traditional concept. *Plant Cell Environ.* 36 1242–1255. 10.1111/pce.12061 23305614

[B58] Kobayashi M. Ohura I. Kawakita K. Yokota N. Fujiwara M. Shimamoto K. (2007). Calcium-dependent protein kinases regulate the production of reactive oxygen species by potato NADPH oxidase. *Plant Cell* 19 1065–1080. 10.1105/tpc.106.048884 17400895 PMC1867354

[B59] Kovtun Y. Chiu W. L. Tena G. Sheen J. (2000). Functional analysis of oxidative stress-activated mitogen-activated protein kinase cascade in plants. *Proc. Natl. Acad. Sci. U.S.A.* 97 2940–2945. 10.1073/pnas.97.6.2940 10717008 PMC16034

[B60] Li C. H. Wang G. Zhao J. L. Zhang L. Q. Ai L. F. Han Y. F. (2014). The receptor-like kinase SIT1 mediates salt sensitivity by activating MAPK3/6 and regulating ethylene homeostasis in rice. *Plant Cell* 26 2538–2553. 10.1105/tpc.114.125187 24907341 PMC4114950

[B61] Li C. R. Liang D. D. Li J. Duan Y. B. Li H. Yang Y. C. (2013). Unravelling mitochondrial retrograde regulation in the abiotic stress induction of rice ALTERNATIVE OXIDASE 1 genes. *Plant Cell Environ.* 36 775–788. 10.1111/pce.12013 22994594

[B62] Li X. Zhang H. Tian L. Huang L. Liu S. Li D. (2015). Tomato SlRbohB, a member of the NADPH oxidase family, is required for disease resistance against Botrytis cinerea and tolerance to drought stress. *Front. Plant Sci.* 6:463 10.3389/fpls.2015.00463 PMC4477072 26157450

[B63] Lin F. Ding H. Wang J. Zhang H. Zhang A. Zhang Y. (2009). Positive feedback regulation of maize NADPH oxidase by mitogen-activated protein kinase cascade in abscisic acid signalling. *J. Exp. Bot.* 60 3221–3238. 10.1093/jxb/erp157 19592501 PMC2718220

[B64] Liu J. Zhou J. Xing D. (2012). Phosphatidylinositol 3-kinase plays a vital role in regulation of rice seed vigor via altering NADPH oxidase activity. *PLoS ONE* 7:e33817 10.1371/journal.pone.0033817 PMC3309022 22448275

[B65] Liu S. Wang M. Wei T. Meng C. Xia G. (2014). A wheat SIMILAR TO RCD-ONE gene enhances seedling growth and abiotic stress resistance by modulating redox homeostasis and maintaining genomic integrity. *Plant Cell* 26 164–180. 10.1105/tpc.113.118687 24443520 PMC3963566

[B66] Lu W. Chu X. Li Y. Wang C. Guo X. (2013). Cotton GhMKK1 induces the tolerance of salt and drought stress, and mediates defence responses to pathogen infection in transgenic *Nicotiana benthamiana*. *PLoS ONE* 8:e68503 10.1371/journal.pone.0068503 PMC3700956 23844212

[B67] Ma F. Lu R. Liu H. Shi B. Zhang J. Tan M. (2012). Nitric oxide-activated calcium/calmodulin-dependent protein kinase regulates the abscisic acid-induced antioxidant defence in maize. *J. Exp. Bot.* 63 4835–4847. 10.1093/jxb/ers161 22865912 PMC3427994

[B68] Marino D. Dunand C. Puppo A. Pauly N. (2012). A burst of plant NADPH oxidases. *Trends Plant Sci.* 17 9–15. 10.1016/j.tplants.2011.10.001 22037416

[B69] Maxwell D. P. Wang Y. Mcintosh L. (1999). The alternative oxidase lowers mitochondrial reactive oxygen production in plant cells. *Proc. Natl. Acad. Sci. U.S.A.* 96 8271–8276. 10.1073/pnas.96.14.8271 10393984 PMC22224

[B70] McCord J. M. Fridovich I. (1969). Superoxide dismutase. An enzymic function for erythrocuprein (hemocuprein). *J. Biol. Chem.* 244 6049–6055. 5389100

[B71] McKersie B. D. Bowley S. R. Harjanto E. Leprince O. (1996). Water-deficit tolerance and field performance of transgenic alfalfa overexpressing superoxide dismutase. *Plant Physiol.* 111 1177–1181. 10.1104/pp.111.4.1177 12226355 PMC160994

[B72] Meyer Y. Belin C. Delorme-Hinoux V. Reichheld J. P. Riondet C. (2012). Thioredoxin and glutaredoxin systems in plants: molecular mechanisms, crosstalks, and functional significance. *Antioxid. Redox Signal.* 17 1124–1160. 10.1089/ars.2011.4327 22531002

[B73] Miller G. Shulaev V. Mittler R. (2008). Reactive oxygen signaling and abiotic stress. *Physiol. Plant.* 133 481–489. 10.1111/j.1399-3054.2008.01090.x 18346071

[B74] Miller G. Suzuki N. Ciftci-Yilmaz S. Mittler R. (2010). Reactive oxygen species homeostasis and signalling during drought and salinity stresses. *Plant Cell Environ.* 33 453–467. 10.1111/j.1365-3040.2009.02041.x 19712065

[B75] Mittal D. Madhyastha D. A. Grover A. (2012). Genome-wide transcriptional profiles during temperature and oxidative stress reveal coordinated expression patterns and overlapping regulons in rice. *PLoS ONE* 7:e40899 10.1371/journal.pone.0040899 PMC3397947 22815860

[B76] Mittler R. (2002). Oxidative stress, antioxidants and stress tolerance. *Trends Plant Sci.* 7 405–410. 10.1016/S1360-1385(02)02312-9 12234732

[B77] Mittler R. Blumwald E. (2010). Genetic engineering for modern agriculture: challenges and perspectives. *Annu. Rev. Plant Biol.* 61 443–462. 10.1146/annurev-arplant-042809-112116 20192746

[B78] Mittler R. Kim Y. Song L. Coutu J. Coutu A. Ciftci-Yilmaz S. (2006). Gain- and loss-of-function mutations in Zat10 enhance the tolerance of plants to abiotic stress. *FEBS Lett.* 580 6537–6542. 10.1016/j.febslet.2006.11.002 17112521 PMC1773020

[B79] Mittler R. Vanderauwera S. Gollery M. Van Breusegem F. (2004). Reactive oxygen gene network of plants. *Trends Plant Sci.* 9 490–498. 10.1016/j.tplants.2004.08.009 15465684

[B80] Mizoi J. Shinozaki K. Yamaguchi-Shinozaki K. (2012). AP2/ERF family transcription factors in plant abiotic stress responses. *Biochim. Biophys. Acta* 1819 86–96. 10.1016/j.bbagrm.2011.08.004 21867785

[B81] Nath K. Kumar S. Poudyal R. S. Yang Y. N. Timilsina R. Park Y. S. (2014). Developmental stage-dependent differential gene expression of superoxide dismutase isoenzymes and their localization and physical interaction network in rice (*Oryza sativa* L.). *Genes Genom.* 36 45–55. 10.1007/s13258-013-0138-9

[B82] Nguyen H. T. Cai S. Jiang G. Ye N. Chu Z. Xu X. (2015). A key ABA catabolic gene, OsABA8ox3, is involved in drought stress resistance in rice. *PLoS ONE* 10:e0116646 10.1371/journal.pone.0116646 PMC4315402 25647508

[B83] Ning J. Li X. Hicks L. M. Xiong L. (2010). A Raf-like MAPKKK gene DSM1 mediates drought resistance through reactive oxygen species scavenging in rice. *Plant Physiol.* 152 876–890. 10.1104/pp.109.149856 20007444 PMC2815886

[B84] Noctor G. Mhamdi A. Foyer C. H. (2014). The roles of reactive oxygen metabolism in drought: not so cut and dried. *Plant Physiol.* 164 1636–1648. 10.1104/pp.113.233478 24715539 PMC3982730

[B85] Oberschall A. Deak M. Torok K. Sass L. Vass I. Kovacs I. (2000). A novel aldose/aldehyde reductase protects transgenic plants against lipid peroxidation under chemical and drought stresses. *Plant J.* 24 437–446. 10.1111/j.1365-313X.2000.00885.x 11115125

[B86] Oda T. Hashimoto H. Kuwabara N. Akashi S. Hayashi K. Kojima C. (2010). Structure of the N-terminal regulatory domain of a plant NADPH oxidase and its functional implications. *J. Biol. Chem.* 285 1435–1445. 10.1074/jbc.M109.058909 19864426 PMC2801269

[B87] Ogasawara Y. Kaya H. Hiraoka G. Yumoto F. Kimura S. Kadota Y. (2008). Synergistic activation of the *Arabidopsis* NADPH oxidase AtrbohD by Ca2+ and phosphorylation. *J. Biol. Chem.* 283 8885–8892. 10.1074/jbc.M708106200 18218618

[B88] Özdemir F. Bor M. Demiral T. Türkan İ (2004). Effects of 24-epibrassinolide on seed germination, seedling growth, lipid peroxidation, proline content and antioxidative system of rice (*Oryza sativa* L.) under salinity stress. *Plant Growth Regul.* 42 203–211. 10.1023/b:grow.0000026509.25995.13

[B89] Perez-Ruiz J. M. Spinola M. C. Kirchsteiger K. Moreno J. Sahrawy M. Cejudo F. J. (2006). Rice NTRC is a high-efficiency redox system for chloroplast protection against oxidative damage. *Plant Cell* 18 2356–2368. 10.1105/tpc.106.041541 16891402 PMC1560923

[B90] Pitzschke A. Djamei A. Bitton F. Hirt H. (2009). A major role of the MEKK1-MKK1/2-MPK4 pathway in ROS signalling. *Mol. Plant* 2 120–137. 10.1093/mp/ssn079 19529823 PMC2639734

[B91] Pitzschke A. Hirt H. (2006). Mitogen-activated protein kinases and reactive oxygen species signaling in plants. *Plant Physiol.* 141 351–356. 10.1104/pp.106.079160 16760487 PMC1475449

[B92] Prashanth S. R. Sadhasivam V. Parida A. (2008). Over expression of cytosolic copper/zinc superoxide dismutase from a mangrove plant *Avicennia marina* in indica rice var Pusa Basmati-1 confers abiotic stress tolerance. *Transgenic Res.* 17 281–291. 10.1007/s11248-007-9099-6 17541718

[B93] Qiao B. Zhang Q. Liu D. Wang H. Yin J. Wang R. (2015). A calcium-binding protein, rice annexin OsANN1, enhances heat stress tolerance by modulating the production of H_2_O_2_. *J. Exp. Bot.* 66 5853–5866. 10.1093/jxb/erv294 26085678

[B94] Raghavendra A. S. Gonugunta V. K. Christmann A. Grill E. (2010). ABA perception and signalling. *Trends Plant Sci.* 15 395–401. 10.1016/j.tplants.2010.04.006 20493758

[B95] Ramegowda V. Senthil-Kumar M. Nataraja K. N. Reddy M. K. Mysore K. S. Udayakumar M. (2012). Expression of a finger millet transcription factor, EcNAC1, in tobacco confers abiotic stress-tolerance. *PLoS ONE* 7:e40397 10.1371/journal.pone.0040397 PMC3394802 22808152

[B96] Rushton P. J. Somssich I. E. Ringler P. Shen Q. J. (2010). WRKY transcription factors. *Trends Plant Sci.* 15 247–258. 10.1016/j.tplants.2010.02.006 20304701

[B97] Sagi M. Davydov O. Orazova S. Yesbergenova Z. Ophir R. Stratmann J. W. (2004). Plant respiratory burst oxidase homologs impinge on wound responsiveness and development in *Lycopersicon esculentum*. *Plant Cell* 16 616–628. 10.1105/tpc.019398 14973161 PMC385276

[B98] Sato Y. Masuta Y. Saito K. Murayama S. Ozawa K. (2011). Enhanced chilling tolerance at the booting stage in rice by transgenic overexpression of the ascorbate peroxidase gene, OsAPXa. *Plant Cell Rep.* 30 399–406. 10.1007/s00299-010-0985-7 21203887

[B99] Schmidt R. Mieulet D. Hubberten H. M. Obata T. Hoefgen R. Fernie A. R. (2013). Salt-responsive ERF1 regulates reactive oxygen species-dependent signaling during the initial response to salt stress in rice. *Plant Cell* 25 2115–2131. 10.1105/tpc.113.113068 23800963 PMC3723616

[B100] Selote D. S. Khanna-Chopra R. (2010). Antioxidant response of wheat roots to drought acclimation. *Protoplasma* 245 153–163. 10.1007/s00709-010-0169-x 20559854

[B101] Setter T. L. Bhekasut P. Greenway H. (2010). Desiccation of leaves after de-submergence is one cause for intolerance to complete submergence of the rice cultivar IR 42. *Funct. Plant Biol.* 37 1096–1104. 10.1071/FP10025

[B102] Shi B. Ni L. Liu Y. Zhang A. Tan M. Jiang M. (2014). OsDMI3-mediated activation of OsMPK1 regulates the activities of antioxidant enzymes in abscisic acid signalling in rice. *Plant Cell Environ.* 37 341–352. 10.1111/pce.12154 23777258

[B103] Shi B. Ni L. Zhang A. Cao J. Zhang H. Qin T. (2012). OsDMI3 is a novel component of abscisic acid signaling in the induction of antioxidant defense in leaves of rice. *Mol. Plant* 5 1359–1374. 10.1093/mp/sss068 22869603

[B104] Shukla D. Huda K. M. Banu M. S. Gill S. S. Tuteja R. Tuteja N. (2014). OsACA6, a P-type 2B Ca2+ ATPase functions in cadmium stress tolerance in tobacco by reducing the oxidative stress load. *Planta* 240 809–824. 10.1007/s00425-014-2133-z 25074587

[B105] Sirichandra C. Gu D. Hu H. C. Davanture M. Lee S. Djaoui M. (2009). Phosphorylation of the *Arabidopsis* AtrbohF NADPH oxidase by OST1 protein kinase. *FEBS Lett.* 583 2982–2986. 10.1016/j.febslet.2009.08.033 19716822

[B106] Sun J. Hu W. Zhou R. Wang L. Wang X. Wang Q. (2015). The *Brachypodium distachyon* BdWRKY36 gene confers tolerance to drought stress in transgenic tobacco plants. *Plant Cell Rep.* 34 23–35. 10.1007/s00299-014-1684-6 25224555

[B107] Sun S. J. Guo S. Q. Yang X. Bao Y. M. Tang H. J. Sun H. (2010). Functional analysis of a novel Cys2/His2-type zinc finger protein involved in salt tolerance in rice. *J. Exp. Bot.* 61 2807–2818. 10.1093/jxb/erq120 20460361 PMC2882275

[B108] Suzuki N. Koussevitzky S. Mittler R. Miller G. (2012). ROS and redox signalling in the response of plants to abiotic stress. *Plant Cell Environ.* 35 259–270. 10.1111/j.1365-3040.2011.02336.x 21486305

[B109] Suzuki N. Miller G. Morales J. Shulaev V. Torres M. A. Mittler R. (2011). Respiratory burst oxidases: the engines of ROS signaling. *Curr. Opin. Plant Biol.* 14 691–699. 10.1016/j.pbi.2011.07.014 21862390

[B110] Takahashi S. Kimura S. Kaya H. Iizuka A. Wong H. L. Shimamoto K. (2012). Reactive oxygen species production and activation mechanism of the rice NADPH oxidase OsRbohB. *J. Biochem.* 152 37–43. 10.1093/jb/mvs044 22528669

[B111] Tang B. Xu S. Z. Zou X. L. Zheng Y. L. Qiu F. Z. (2010). Changes of antioxidative enzymes and lipid peroxidation in leaves and roots of waterlogging-tolerant and waterlogging-sensitive maize genotypes at seedling stage. *Agric. Sci. China* 9 651–661. 10.1016/S1671-2927(09)60140-1

[B112] Teixeira F. K. Menezes-Benavente L. Galvao V. C. Margis R. Margis-Pinheiro M. (2006). Rice ascorbate peroxidase gene family encodes functionally diverse isoforms localized in different subcellular compartments. *Planta* 224 300–314. 10.1007/s00425-005-0214-8 16397796

[B113] Teixeira F. K. Menezes-Benavente L. Margis R. Margis-Pinheiro M. (2004). Analysis of the molecular evolutionary history of the ascorbate peroxidase gene family: inferences from the rice genome. *J. Mol. Evol.* 59 761–770. 10.1007/s00239-004-2666-z 15599508

[B114] Torres M. A. Dangl J. L. (2005). Functions of the respiratory burst oxidase in biotic interactions, abiotic stress and development. *Curr. Opin. Plant Biol.* 8 397–403. 10.1016/j.pbi.2005.05.014 15939662

[B115] Turan O. Ekmekci Y. (2011). Activities of photosystem II and antioxidant enzymes in chickpea (*Cicer arietinum* L.) cultivars exposed to chilling temperatures. *Acta Physiol. Plant.* 33 67–78. 10.1007/s11738-010-0517-7

[B116] Tuteja N. Sahoo R. K. Garg B. Tuteja R. (2013). OsSUV3 dual helicase functions in salinity stress tolerance by maintaining photosynthesis and antioxidant machinery in rice (*Oryza sativa* L. cv. IR64). *Plant J.* 76 115–127. 10.1111/tpj.12277 23808500

[B117] Ulker B. Somssich I. E. (2004). WRKY transcription factors: from DNA binding towards biological function. *Curr. Opin. Plant Biol.* 7 491–498. 10.1016/j.pbi.2004.07.012 15337090

[B118] Wang B. Q. Zhang Q. F. Liu J. H. Li G. H. (2011). Overexpression of PtADC confers enhanced dehydration and drought tolerance in transgenic tobacco and tomato: effect on ROS elimination. *Biochem. Biophys. Res. Commun.* 413 10–16. 10.1016/j.bbrc.2011.08.015 21871871

[B119] Wang F. Chen H. W. Li Q. T. Wei W. Li W. Zhang W. K. (2015). GmWRKY27 interacts with GmMYB174 to reduce expression of GmNAC29 for stress tolerance in soybean plants. *Plant J.* 83 224–236. 10.1111/tpj.12879 25990284

[B120] Wang G. F. Li W. Q. Li W. Y. Wu G. L. Zhou C. Y. Chen K. M. (2013). Characterization of rice NADPH oxidase genes and their expression under various environmental conditions. *Int. J. Mol. Sci.* 14 9440–9458. 10.3390/ijms14059440 23629674 PMC3676792

[B121] Wen F. Qin T. Wang Y. Dong W. Zhang A. Tan M. (2015). OsHK3 is a crucial regulator of abscisic acid signaling involved in antioxidant defense in rice. *J. Integr. Plant Biol.* 57 213–228. 10.1111/jipb.12222 24912543

[B122] Wong H. L. Pinontoan R. Hayashi K. Tabata R. Yaeno T. Hasegawa K. (2007). Regulation of rice NADPH oxidase by binding of Rac GTPase to its N-terminal extension. *Plant Cell* 19 4022–4034. 10.1105/tpc.107.055624 18156215 PMC2217649

[B123] Wu L. Zhang Z. Zhang H. Wang X. C. Huang R. (2008). Transcriptional modulation of ethylene response factor protein JERF3 in the oxidative stress response enhances tolerance of tobacco seedlings to salt, drought, and freezing. *Plant Physiol.* 148 1953–1963. 10.1104/pp.108.126813 18945933 PMC2593663

[B124] Xia X. J. Gao C. J. Song L. X. Zhou Y. H. Shi K. Yu J. Q. (2014). Role of H_2_O_2_ dynamics in brassinosteroid-induced stomatal closure and opening in *Solanum lycopersicum*. *Plant Cell Environ.* 37 2036–2050. 10.1111/pce.12275 24428600

[B125] Xia X. J. Wang Y. J. Zhou Y. H. Tao Y. Mao W. H. Shi K. (2009). Reactive oxygen species are involved in brassinosteroid-induced stress tolerance in cucumber. *Plant Physiol.* 150 801–814. 10.1104/pp.109.138230 19386805 PMC2689980

[B126] Xue T. Li X. Zhu W. Wu C. Yang G. Zheng C. (2009). Cotton metallothionein GhMT3a, a reactive oxygen species scavenger, increased tolerance against abiotic stress in transgenic tobacco and yeast. *J. Exp. Bot.* 60 339–349. 10.1093/jxb/ern291 19033550 PMC3071772

[B127] Yamaguchi-Shinozaki K. Shinozaki K. (2006). Transcriptional regulatory networks in cellular responses and tolerance to dehydration and cold stresses. *Annu. Rev. Plant Biol.* 57 781–803. 10.1146/annurev.arplant.57.032905.105444 16669782

[B128] Yan H. Jia H. Chen X. Hao L. An H. Guo X. (2014). The cotton WRKY transcription factor GhWRKY17 functions in drought and salt stress in transgenic *Nicotiana benthamiana* through ABA signaling and the modulation of reactive oxygen species production. *Plant Cell Physiol.* 55 2060–2076. 10.1093/pcp/pcu133 25261532

[B129] Yan J. Guan L. Sun Y. Zhu Y. Liu L. Lu R. (2015). Calcium and ZmCCaMK are involved in brassinosteroid-induced antioxidant defense in maize leaves. *Plant Cell Physiol.* 56 883–896. 10.1093/pcp/pcv014 25647327

[B130] Yang C. J. Zhang C. Lu Y. N. Jin J. Q. Wang X. L. (2011). The mechanisms of brassinosteroids’ action: from signal transduction to plant development. *Mol. Plant* 4 588–600. 10.1093/mp/ssr020 21471332

[B131] Yang Z. Wu Y. Li Y. Ling H. Q. Chu C. (2009). OsMT1a, a type 1 metallothionein, plays the pivotal role in zinc homeostasis and drought tolerance in rice. *Plant Mol. Biol.* 70 219–229. 10.1007/s11103-009-9466-1 19229638

[B132] Ye N. Zhu G. Liu Y. Li Y. Zhang J. (2011). ABA controls H_2_O_2_ accumulation through the induction of OsCATB in rice leaves under water stress. *Plant Cell Physiol.* 52 689–698. 10.1093/pcp/pcr028 21398647

[B133] Yoshioka H. Numata N. Nakajima K. Katou S. Kawakita K. Rowland O. (2003). *Nicotiana benthamiana* gp91phox homologs NbrbohA and NbrbohB participate in H_2_O_2_ accumulation and resistance to *Phytophthora infestans*. *Plant Cell* 15 706–718. 10.1105/tpc.008680 12615943 PMC150024

[B134] You J. Hu H. Xiong L. (2012). An ornithine delta-aminotransferase gene OsOAT confers drought and oxidative stress tolerance in rice. *Plant Sci.* 197 59–69. 10.1016/j.plantsci.2012.09.002 23116672

[B135] You J. Zong W. Hu H. Li X. Xiao J. Xiong L. (2014). A STRESS-RESPONSIVE NAC1-regulated protein phosphatase gene rice protein phosphatase18 modulates drought and oxidative stress tolerance through abscisic acid-independent reactive oxygen species scavenging in rice. *Plant Physiol.* 166 2100–2114. 10.1104/pp.114.251116 25318938 PMC4256856

[B136] You J. Zong W. Li X. Ning J. Hu H. Xiao J. (2013). The SNAC1-targeted gene OsSRO1c modulates stomatal closure and oxidative stress tolerance by regulating hydrogen peroxide in rice. *J. Exp. Bot.* 64 569–583. 10.1093/jxb/ers349 23202132 PMC3542048

[B137] Zhang A. Jiang M. Zhang J. Ding H. Xu S. Hu X. (2007). Nitric oxide induced by hydrogen peroxide mediates abscisic acid-induced activation of the mitogen-activated protein kinase cascade involved in antioxidant defense in maize leaves. *New Phytol.* 175 36–50. 10.1111/j.1469-8137.2007.02071.x 17547665

[B138] Zhang A. Jiang M. Zhang J. Tan M. Hu X. (2006). Mitogen-activated protein kinase is involved in abscisic acid-induced antioxidant defense and acts downstream of reactive oxygen species production in leaves of maize plants. *Plant Physiol.* 141 475–487. 10.1104/pp.105.075416 16531486 PMC1475456

[B139] Zhang A. Zhang J. Ye N. Cao J. Tan M. Jiang M. (2010). ZmMPK5 is required for the NADPH oxidase-mediated self-propagation of apoplastic H_2_O_2_ in brassinosteroid-induced antioxidant defence in leaves of maize. *J. Exp. Bot.* 61 4399–4411. 10.1093/jxb/erq243 20693409 PMC2955750

[B140] Zhang C. J. Zhao B. C. Ge W. N. Zhang Y. F. Song Y. Sun D. Y. (2011). An apoplastic h-type thioredoxin is involved in the stress response through regulation of the apoplastic reactive oxygen species in rice. *Plant Physiol.* 157 1884–1899. 10.1104/pp.111.182808 22010108 PMC3327207

[B141] Zhang H. Liu Y. Wen F. Yao D. Wang L. Guo J. (2014). A novel rice C2H2-type zinc finger protein, ZFP36, is a key player involved in abscisic acid-induced antioxidant defence and oxidative stress tolerance in rice. *J. Exp. Bot.* 65 5795–5809. 10.1093/jxb/eru313 25071223 PMC4203119

[B142] Zhang H. Ni L. Liu Y. Wang Y. Zhang A. Tan M. (2012a). The C2H2-type zinc finger protein ZFP182 is involved in abscisic acid-induced antioxidant defense in rice. *J. Integr. Plant Biol.* 54 500–510. 10.1111/j.1744-7909.2012.01135.x 22693960

[B143] Zhang L. Li Y. Lu W. Meng F. Wu C. A. Guo X. (2012b). Cotton GhMKK5 affects disease resistance, induces HR-like cell death, and reduces the tolerance to salt and drought stress in transgenic *Nicotiana benthamiana*. *J. Exp. Bot.* 63 3935–3951. 10.1093/jxb/ers086 22442420 PMC3388830

[B144] Zhang Q. (2007). Strategies for developing Green Super Rice. *Proc. Natl. Acad. Sci. U.S.A.* 104 16402–16409. 10.1073/pnas.0708013104 17923667 PMC2034246

[B145] Zhang Y. Tan J. Guo Z. Lu S. He S. Shu W. (2009). Increased abscisic acid levels in transgenic tobacco over-expressing 9 cis-epoxycarotenoid dioxygenase influence H_2_O_2_ and NO production and antioxidant defences. *Plant Cell Environ.* 32 509–519. 10.1111/j.1365-3040.2009.01945.x 19183289

[B146] Zhang Z. Zhang Q. Wu J. Zheng X. Zheng S. Sun X. (2013). Gene knockout study reveals that cytosolic ascorbate peroxidase 2 (OsAPX2) plays a critical role in growth and reproduction in rice under drought, salt and cold stresses. *PLoS ONE* 8:e57472 10.1371/journal.pone.0057472 PMC3585366 23468992

[B147] Zhou J. Wang J. Li X. Xia X. J. Zhou Y. H. Shi K. (2014a). H_2_O_2_ mediates the crosstalk of brassinosteroid and abscisic acid in tomato responses to heat and oxidative stresses. *J. Exp. Bot.* 65 4371–4383. 10.1093/jxb/eru217 24899077 PMC4112640

[B148] Zhou T. Yang X. Wang L. Xu J. Zhang X. (2014b). GhTZF1 regulates drought stress responses and delays leaf senescence by inhibiting reactive oxygen species accumulation in transgenic *Arabidopsis*. *Plant Mol. Biol.* 85 163–177. 10.1007/s11103-014-0175-z 24473898

[B149] Zhu J. Y. Sae-Seaw J. Wang Z. Y. (2013a). Brassinosteroid signalling. *Development* 140 1615–1620. 10.1242/dev.060590 23533170 PMC3621480

[B150] Zhu Y. Zuo M. Liang Y. Jiang M. Zhang J. Scheller H. V. (2013b). MAP65-1a positively regulates H_2_O_2_ amplification and enhances brassinosteroid-induced antioxidant defence in maize. *J. Exp. Bot.* 64 3787–3802. 10.1093/jxb/ert215 23956414 PMC3745737

